# Impact of empiric antibiotics on risk of *Clostridioides difficile*—a causal inference observational analysis

**DOI:** 10.1017/ash.2025.10

**Published:** 2025-02-12

**Authors:** Matthew A. Pappas, Shoshana J. Herzig, Andrew D. Auerbach, Abhishek Deshpande, Eunice Blanchard, Michael B. Rothberg

**Affiliations:** 1 Department of Hospital Medicine, Cleveland Clinic, Cleveland, OH, USA; 2 Center for Value-Based Care Research, Cleveland Clinic, Cleveland, OH, USA; 3 COVID-19 Consortium of HCA Healthcare and Academia for Research Generation, Nashville, TN, USA; 4 Department of Medicine, Division of General Medicine, and Harvard Medical School, Beth Israel Deaconess Medical Center, Boston, MA, USA; 5 Department of Hospital Medicine, University of California, San Francisco, CA, USA; 6 Infection Control and Hospital Epidemiology, HCA Healthcare, Nashville, TN, USA

## Abstract

**Background:**

*Clostridioides difficile* infection (CDI) is a common and often nosocomial infection associated with increased mortality and morbidity. Antibiotic use is the most important modifiable risk factor, but many patients require empiric antibiotics. We estimated the increased risk of hospital-onset CDI with one daily dose-equivalent (DDE) of various empiric antibiotics compared to management without that daily dose-equivalent.

**Methods:**

Using a multicenter retrospective cohort of adults admitted between March 2, 2020 and February 11, 2021 for the treatment of SARS-CoV-2, we used a series of three-level logistic regression models to estimate the probability of receiving each of several antibiotics of interest. For each antibiotic, we then limited our data set to patient-days at intermediate probability of receipt and used augmented inverse-probability weighted models to estimate the average treatment effect of one daily dose-equivalent, compared to management without that daily dose-equivalent, on the probability of hospital-onset CDI.

**Results:**

In 24,406 patient-days at intermediate probability of receipt, parenteral vancomycin increased risk of hospital-onset CDI, with an average treatment effect of 0.0096 cases per daily dose-equivalent (95% CI: 0.0053—0.0138). In 38,003 patient-days at intermediate probability of receipt, cefepime also increased subsequent CDI risk, with an estimated effect of 0.0074 more cases per daily dose-equivalent (95% CI: 0.0022—0.0126).

**Conclusions:**

Among common empiric antibiotics, parenteral vancomycin and cefepime appeared to increase risk of hospital-onset CDI. Causal inference observational study designs can be used to estimate patient-level harms of interventions such as empiric antimicrobials.

## Introduction

*Clostridioides difficile* infection (CDI, formerly *Clostridium difficile* infection) is a common and often healthcare-associated infection associated with increased mortality, morbidity, and cost.^1–3^ Many antibiotics are associated with both initial and recurrent CDI; antibacterial use may be the most important modifiable risk factor.^4–8^

Observational analyses of antibiotic use and risk of CDI are subject to multiple important biases. First, present-on-admission codes are unreliable; it is therefore difficult to know from administrative data whether CDI was a reason for admission or acquired during the hospitalization.^9^ Second, physician selection of antibiotics may depend on such factors as acuity of illness and perceived risk of CDI, which confound apparent relationships between antibiotic use and subsequent CDI. Third, the distinction between colonization and infection remains imprecise, while diarrhea is a common side effect of many antibiotics.^10^ As such, physicians may ascribe benign diarrhea to CDI among colonized patients who received an antibiotic they believe to confer additional risk.

For patients who are given empiric antibiotics, which antibiotic would confer the lowest increase in a patient’s risk of healthcare-associated CDI remains an important and unanswered question. Using a cohort of patients hospitalized for COVID-19 before the widespread availability of vaccines, we used a causal inference observational approach to estimate the effect of one day of different antibiotics commonly given as empiric therapy on subsequent risk of hospital-onset CDI.

## Methods

### Patient population and characteristics

HCA Healthcare is a large for-profit healthcare corporation that operated 186 hospitals in the United States in 2020 and 2021. These hospitals are predominantly located in the southern United States, and the majority are medium-to-large community hospitals. Electronic health record (EHR) data from HCA facilities are centralized into a data warehouse. Through the Consortium for HCA Healthcare and Academia Research GEneration (CHARGE), HCA made data available from a multicenter retrospective observational cohort of adults 18 years of age or older who were admitted to any HCA hospital in the U.S. between March 2, 2020 and February 11, 2021 for the treatment of SARS-CoV-2. The cohort development has been described previously.^11,12^

From the CHARGE cohort, we included each patient’s first hospitalization for treatment of COVID-19. Present-on-admission codes are unlikely to have acceptable accuracy in this data set; we therefore excluded hospitalizations with any doses of fidaxomicin, oral vancomycin, or metronidazole in the first 48 hours of hospitalization, as these patients could plausibly have had CDI on presentation.^9,13^ We also excluded any hospitalizations shorter than 48 hours. As sensitivity analyses, we used 24 and 72 hours as the time of inclusion/exclusion as well. Our analyses included all patient-days after inclusion/exclusion without discharge or a previous diagnosis of healthcare-associated CDI.

### Outcome and exposures

Our outcome was a diagnosis of CDI during the index hospitalization, identified by the combination of an appropriate ICD-10 code (A01.71 or A04.72) and receipt of one or more doses of a possible treatment for CDI (metronidazole, oral vancomycin, and/or fidaxomicin) before hospital discharge, an approach that should be highly accurate despite our lack of testing data.^14^ The first date of treatment was taken to be the date of CDI onset. Patient-days after diagnosis and treatment of CDI were excluded.

We considered as exposures several antibiotics commonly used in the care of patients with pulmonary infections (COVID-19 and others): azithromycin, ceftriaxone, cefepime, levofloxacin, piperacillin/tazobactam, vancomycin, and meropenem. On each patient-day, we identified whether at least one daily-dose equivalent of each antibiotic was administered. We calculated daily dose-equivalents based on 24-hours of therapy using common inpatient doses. These differ somewhat from WHO’s defined daily dose (DDD); for example, a daily dose-equivalent of vancomycin would vary depending on a patient’s weight and we considered 1g of ceftriaxone to be treatment, rather than only 2g. Further detail can be found in the Supplemental Appendix.

### Analyses and covariates

We theorized that: (1) antibiotics would be more likely to be given to patients who were older, less clinically stable, or who had received the same antibiotic the previous day, (2) patients would differ in likelihood of receiving each antibiotic, and (3) the tendency to administer antimicrobials would differ across hospitals. We therefore created, for each antibiotic studied, a three-level logistic model to estimate the probability of antibiotic receipt on a given patient-day, with patient-days clustered by patients and patients clustered within hospitals. We included temperature, systolic blood pressure, heart rate, respiratory rate, white blood cell (WBC) count, the number of days since admission, and whether the patient had been administered the antibiotic of interest on the previous day as covariates at the patient-day level and age and sex at the patient level. Vital signs and labs were averaged on each calendar day. We treated each continuous variable as a restricted cubic spline to allow for nonlinear effects.^15^ Physicians might also alter antibiotic choice for patients admitted from a nursing facility (for example, due to resistance patterns of colonizing organisms); we therefore added whether the patient was admitted from a nursing facility as a patient-level predictor. A directed acyclic graph (DAG) is included in the Supplemental Appendix. We used multiple imputation with chained equations to reduce bias from missing data and the White, Royston, and Wood guidelines to ensure imputation did not introduce Monte Carlo error.^16^ We ensured adequate calibration of each antibiotic’s model using the Brier score.^17^ Detailed information regarding these steps can be found in the Supplemental Appendix.

For each antibiotic, we then used augmented inverse probability weighting (AIPW) to control for both the probability of antibiotic receipt and the probability of CDI. This “doubly-robust” approach may produce accurate estimates even if one of the two models is misspecified, and therefore reduce sensitivity of results to model specification.^18,19^ To estimate the probability of antibiotic receipt, we used the three-level models described above. In our AIPW outcome model, we included age, sex, and whether the patient was admitted from a nursing facility as covariates. To avoid extreme weights, we trimmed patient-days where the estimated probability of antibiotic receipt was either less than 5% or greater than 95% for each antibiotic, and ensured remaining patient-days were balanced with respect to probability of receipt by visual inspection of weighted density plots. If necessary to achieve model convergence or appropriate propensity weighting overlap, we trimmed additional days from the analysis in increments of 1%. When doing so, we also verified that the outcome model retained at least 10 outcomes per included predictor. Overlap plots are included in the Supplemental Appendix.

Finally, to estimate how robust our findings would be to unmeasured confounding, we computed E-values for each antibiotic with a nonnull risk difference in our causal inference models.^20^

As a post-hoc analysis to better understand an unexpected result, we also compared receipt of one dose-equivalent of azithromycin against no antibiotics at all. Details of this analysis are included in the Supplemental Appendix.

Analyses were approved by the Cleveland Clinic Institutional Review Board and performed in Stata (version 16.2, College Station, TX).

## Results

Among 99,114 adults, we identified 104,647 hospital admissions. We excluded 13,485 hospitalizations of 48 hours or less. Among hospitalizations longer than 48 hours, we excluded 2,142 in which the patient received one or more potential treatments for CDI in the first 48 hours: 1,616 received metronidazole, 602 received oral vancomycin, and 2 received fidaxomicin. Our resulting sample included 91,411 index hospitalizations for COVID-19. Patient and hospitalization characteristics are shown in Table 1.

**Table 1.** Patient and hospitalization characteristics

Variable	Not diagnosed with or not treated for inpatient CDI	Diagnosed with and treated for inpatient CDI	Overall
	(N = 91,022)	(N = 128)	(N = 91,150)
Age in years, median (IQR)	67 (54–77)	74 (64–82)	67 (54–77)
Male sex (%)	53.4%	45.3%	53.4%
Patient state of residence			
Texas	27,115	31	27,146
Florida	26,616	43	26,659
California	5,198	10	5,208
Georgia	4,891	6	4,897
Tennessee	4,744	9	4,753
Nevada	4,714	8	4,722
Other	17,744	21	17,765
Admitted from nursing facility (%)	3.2%	6.3%	3.2%
Taking proton-pump inhibitor before admission (%)	11.9%	12.5%	11.9%
Length of stay, median (IQR)	7 (4–12)	10 (6–18)	7 (4–12)
Received at least one day of azithromycin	31.9%	16.9%	31.9%
Received at least one day of ceftriaxone	32.6%	34.7%	32.6%
Received at least one day of cefepime	6.4%	24.2%	6.5%
Received at least one day of piperacillin/tazobactam	4.4%	10.5%	4.5%
Received at least one day of meropenem	1.0%	0.0%	1.0%
Received at least one day of levofloxacin	1.2%	2.4%	1.2%
Received at least one day of IV vancomycin	4.9%	43.5%	5.0%

A total of 128 hospitalizations (0.13%) included diagnosis and treatment of CDI over a total of 922,187 patient-days, for an incidence rate of 1.4 cases per 10,000 patient-days at risk.

The most prescribed antibiotic in this cohort was ceftriaxone (24.9% of patient-days), followed by azithromycin (23.8%). Other antibiotics included cefepime (7.3% of patient-days), vancomycin (6.1%), piperacillin/tazobactam (5.7%), meropenem (2.0%), and levofloxacin (1.1%). Around 53.2% of patient-days did not include a daily dose-equivalent of any of our antibiotics of interest. Our multilevel logistic regression models for each antibiotic demonstrated reasonable discrimination and calibration, with C-statistics ranging from 0.85 (vancomycin) to 0.94 (meropenem) and Brier scores ranging from 0.01 (levofloxacin) to 0.10 (ceftriaxone). Additional information regarding each model, including calibration plots, are included in the Supplemental Appendix.

After trimming extreme probabilities, too few cases of CDI remained among patients given meropenem or levofloxacin to fit causal inference models. Numbers of patient-days and summary statistics for patient-days included in our causal inference models are shown in Table 2.

**Table 2.** Characteristics of patient-days included in analyses

	Azithromycin		Ceftriaxone		Cefepime		Piperacillin/tazobactam		Parenteral vancomycin	
	Not given	Given	Not given	Given	Not given	Given	Not given	Given	Not given	Given
Number of patient-days	23,561	23,352	24,641	25,008	28,973	2,262	24,143	2,030	16,544	2,718
Age in years, median (IQR)	67 (54–78)	65 (52–76)	67 (54–78)	66 (53–77)	67 (55–78)	71 (59–80)	68 (56–78)	70 (57–79)	67 (55–77)	70 (58–80)
Male sex (%)	52.2%	53.0%	53.3%	52.5%	54.9%	53.4%	59.1%	58.1%	58.1%	56.5%
Admitted from nursing facility (%)	3.3%	2.3%	3.1%	2.3%	3.5%	7.6%	3.9%	5.4%	3.3%	6.8%
Received antibiotic on previous day (%)	21.9%	59.7%	24.1%	60.1%	1.2%	43.0%	1.8%	66.8%	7.6%	58.4%
Temperature (°C), median (IQR)	36.8 (36.6–37.1)	36.8 (36.6–37.0)	36.8 (36.6–37.0)	36.8 (36.6–37.0)	36.8 (36.6–37.1)	36.8 (36.6–37.1)	36.8 (36.6–37.2)	36.7 (36.5–37.0)	36.9 (36.6–37.4)	36.8 (36.6–37.2)
White blood cell count, median (IQR)	7.8 (5.5–11.2)	7.8 (5.6–11.0)	8.2 (5.6–11.8)	7.9 (5.6–11.2)	9.4 (6.6–12.7)	7.5 (5.3-10.3)	11.6 (7.9–15.8)	9.7 (6.9–13.3)	9.4 (6.4–13.9)	10.4 (6.9–15.7)
Systolic blood pressure (mmHg), median (IQR)	129 (117–143)	129 (118–143)	128 (116–141)	129 (118–143)	128 (117–142)	129 (116–143)	125 (114–139)	128 (116–142)	126 (114–140)	125 (112–139)
Respiratory rate, median (IQR)	18 (17–21)	18 (17–20)	18 (17–22)	18 (17–20)	19 (17–22)	18 (17–20)	22 (18–26)	18 (17–21)	24 (19–28)	20 (18–23)
Heart rate, median (IQR)	80 (70–91)	78 (69–89)	80 (70–91)	79 (70–90)	81 (71–91)	81 (72-92)	84 (74–95)	80 (70–91)	86 (75–98)	84 (73–97)

Parenteral vancomycin was associated with an absolute increase of 0.0096 cases of hospital-onset CDI per daily dose-equivalent (95%CI: 0.0053–0.0138). Each daily dose-equivalent of cefepime was associated with 0.0074 more cases of hospital-onset CDI (95%CI: 0.0022–0.0126). The effect of piperacillin/tazobactam did not differ from the null. Azithromycin was associated with 0.0027 *fewer* cases of hospital-onset CDI per daily dose-equivalent administered (95%CI: 0.0036–0.0018). Absolute risk differences with receipt of one daily-dose equivalent of each antibiotic are shown in Table 3. These results were robust to inclusion/exclusion after 24 or 72 hours of admission. Ceftriaxone did not differ from the null in our base-case analysis, but this estimate was more sensitive to the time of inclusion/exclusion. Results of both sensitivity analyses are included in the appendix.

**Table 3.** Absolute treatment effect of one daily dose-equivalent of each antibiotic on the rate of hospital-onset *C. difficile* infection

	Absolute risk increase	95% CI		p	Mean NNH
Azithromycin	−0.0027	−0.0036	−0.0018	<0.001	–
Ceftriaxone	−0.0005	−0.0015	0.0005	>0.2	–
Cefepime	0.0074	0.0022	0.0126	0.006	135
Piperacillin/tazobactam	0.0025	−0.0020	0.0070	>0.2	399
Vancomycin	0.0096	0.0053	0.0138	<0.001	105

Calculated as relative rather than absolute effects, azithromycin was associated with a relative risk of CDI of 0.17 (95% CI: 0.10–0.29), ceftriaxone was associated with a relative risk of CDI of 0.61 (95% CI: 0.36–1.03), cefepime was associated with a relative risk of CDI of 4.62 (95% CI: 2.20–9.71), piperacillin/tazobactam was associated with a relative risk of CDI of 2.24 (95% CI: 0.78–6.43), and vancomycin was associated with a relative risk of CDI of 7.13 (95% CI: 4.25–11.95).

In our post-hoc analysis, receipt of azithromycin did not appear to change the rate of subsequent CDI compared to the counterfactual of receiving no doses of other considered antibiotics, with an absolute treatment effect of 2.6e-06 fewer cases per daily dose-equivalent (*P* > 0.2, 95% CI: -0.0001 to 0.0001).

For an unmeasured confounder to shift the observed risk difference from one daily dose-equivalent of vancomycin to a null effect, its E-value would have to be 13.6. That is, a confounder that was associated with both vancomycin administration and CDI by a risk ratio of 13.6-fold each could explain away this effect, while a weaker confounder could not. The corresponding E-value for cefepime was 9.6.

## Discussion

Antibiotic use is likely the most important modifiable risk factor for *Clostridioides difficile* infection. Previous observational analyses have measured the association between different antibiotics and risk of *C. difficile* colonization, infection, and recurrent infection.^4,6,21–24^ In this study, we used causal inference observational models to estimate the effect of one daily-dose equivalent of each of several common empiric antibiotics, compared to not administering one daily dose of that antibiotic.

Prompt broad-spectrum antibiotics can improve survival in severely ill patients.^25^ However, antimicrobials have untoward effects as well, which must prompt stewardship and deescalation. Appropriately balancing the benefits and harms of antibiotics to optimally deescalate empiric therapy would require clear estimates of patient-level harms and societal externalities, not simply the risk of hospital-onset CDI. Still, we hope that our precise estimates of one adverse consequence can inform future decision analyses.

This analysis offers several advantages over previous observational work. First, all patients were admitted for a distinct reason of acute onset (COVID-19). Few patients would be expected to have acute onset of CDI and COVID-19 simultaneously in this epoch of COVID-19; after excluding patients who received possible CDI treatments in the first 48 hours of hospitalization, our cohort should contain almost no patients admitted for CDI. Second, due to early COVID-19 precautions, isolation procedures likely exceeded those used in most other times and places.^26^ If non-pharmaceutical hospital care affects risk of CDI, as is sometimes theorized, this data set allows a clearer estimate of the effect of antibiotics alone. Third, we used causal inference models to minimize confounding by indication. To the extent that the decision to administer each antibiotic (compared to alternatives without that antibiotic) can be modeled based using variables captured in our data set, our use of causal effect models should minimize this critical bias. Fourth, all patients in our cohort were admitted for a viral infection which is not improved by any antibiotics and which has low rates of bacterial coinfection.^27–29^ This allows more confidence in causal exchangeability: after controlling for probability of antibiotic receipt, patient outcomes (including rates of CDI) should be substantially similar in patients who received antibiotics as those who did not.

To parallel the decision faced by clinicians considering initiating or discontinuing empiric antibiotics, each of our causal inference models compares one daily dose-equivalent against management without that antibiotic, rather than comparing against no antibiotics whatsoever. However, this means the results for each antibiotic are not directly comparable. A physician considering cefepime had different available alternatives than a physician considering azithromycin or vancomycin. In our primary analysis, azithromycin appeared to reduce the risk of CDI, which is discordant with theory and observational analyses.^22,30^ In our post-hoc analysis comparing azithromycin to no antibiotics, azithromycin did not affect risk of CDI. Physicians who prescribed azithromycin may have done so in lieu of higher-risk alternative coverage of atypical organisms (including, for example, fluoroquinolones), leading to an apparently lower risk when physicians chose azithromycin. In other words, the mechanism of this apparent risk decrease may be antimicrobial substitution rather than the macrolide itself.

Unlike other empiric antibiotics in our study, physicians would have lacked a clear alternative to vancomycin. Our data were not adequate to test other antibiotics used for coverage of methicillin-resistant Staph aureus (MRSA), such as daptomycin, linezolid, or ceftaroline; that vancomycin confers the highest risk of CDI in this study could reflect the lack of an alternative. Replacing empiric vancomycin treatment with an antimicrobial of similar spectrum may be ineffective or counterproductive. Clindamycin in particular seems likely to confer even higher risk than vancomycin.^31^ If physicians wish to minimize the risk of hospital-onset CDI from empiric antibiotics, a patient’s risk of infection with MRSA is a key decision parameter.

Previous observational analyses have found associations between vancomycin and CDI, and our study furthers a hypothesis that parenteral vancomycin is causally related to CDI.^30,32^ Previously hypothesized mechanisms include excretion of vancomycin in stool at concentrations adequate to disrupt colonic flora but inadequate for treatment of *C. difficile*.^32^ It is also possible that vancomycin (or any of the other antibiotics we studied) cause CDI in a practical sense, but not in a biologic sense.^33^ Antibiotics commonly cause loose stools, which could precipitate testing for and diagnosis of CDI in colonized persons. If future analyses demonstrated that specific antibiotics increase *C. difficile* testing with equivalent rates of diagnosis and treatment, those antibiotics could be thought of as causing inpatient identification of *C. difficile* carriage rather than causing CDI.^10^ Such an approach would be similar to work on “epidemiologic signatures” described in other settings.^34^

As with all observational studies, our data set is likely to have unmeasured confounding. However, E-value calculations argue against unmeasured confounding as a primary explanation of our results. To shift our estimated risk difference for one daily dose-equivalent of vancomycin to a null effect, an unmeasured confounder would have to be associated with both vancomycin administration and hospital-onset CDI by 13.6-fold. Meanwhile, our conclusion that cefepime increases the risk of hospital-onset CDI would be vulnerable to an unmeasured confounder that had a 9.6-fold association with both cefepime administration and CDI. Unmeasured confounders with risk ratios lower than those would not change the conclusions that each of those antibiotics increases the risk of CDI.

Other limitations remain. Our data set lacks CDI test results; a data set with CDI testing could address time-dependent confounding through daily probability of testing, further unraveling the causal pathway between specific antibiotics and CDI.^35^ Also, widespread isolation precautions and the gastrointestinal manifestations of COVID-19 may have changed testing practices in this cohort and/or changed the probability of new exposure to *C. difficile*.^36^ While that allows a more precise estimate of the effects of antimicrobials, the distinctive features of inpatient care in this period may limit generalizability to other cohorts. We lacked some common measures of nosocomial infection, such as colonization pressure. The high mortality of this condition may have led fewer future cases of CDI to become apparent. We investigated hospital-onset CDI, not cases that arise following discharge. Finally, we considered each daily dose-equivalent of each antibiotic to be independent of other doses of that antibiotic and doses of other medications, such as other antibiotics and acid-suppressive therapies. If multiple doses of one antibiotic have a more-than-additive effect, or if other medications (including antimicrobials) modify the effect of the antibiotics we considered, our estimated effect of one daily dose-equivalent would be a simplistic representation of reality.

Despite these limitations, we believe these analyses further our understanding of causal relationships between empiric antibiotics and CDI. For an outcome as rare as CDI, a randomized clinical trial would likely be impracticable even for the most common antimicrobials in clinical use. Where randomized trial data are lacking, causal inference observational analyses like this one can further the argument for causal relationships.^37–39^ In other recent work, methods similar to ours have produced results similar to a randomized trial.^40^ In hopes of informing clinical practice, we explicitly designed our analyses to reflect the consequences of the decision faced in clinical practice for hospitalized patients: whether or not to administer a chosen antibiotic for an acutely ill patient on a given day.

In summary, we used a large cohort of patients hospitalized for care of COVID-19, many of whom were given empiric antibiotics, to study the causal effect of different antibiotics on risk of hospital-onset *C. difficile* infection. Parenteral vancomycin and cefepime each appeared to increase the risk of hospital-onset CDI. When the risk of MRSA infection is low, stewardship of vancomycin without substitution by other MRSA-covering agents may be the best strategy to reduce hospital-onset CDI. Efforts to reduce hospital-onset CDI may wish to focus on identifying colonized persons, clarifying the distinction between CDI colonization and infection, and identifying persons for whom MRSA is a likely pathogen.

## Supporting information

Pappas et al. supplementary material Pappas et al. supplementary material

## Supplementary material

For supplementary material accompanying this paper visit http://doi.org/10.1017/ash.2025.10. click here to view supplementary material
