# Supplementary material for: Impact of empiric antibiotics on risk of Clostridioides difficile—a causal inference observational analysis
Source: Antimicrob Steward Healthc Epidemiol. 2025 Feb 12;5(1):e40. doi: 10.1017/ash.2025.10 (PMC11822578; doi:10.1017/ash.2025.10)
Supplement: Pappas et al. supplementary material [file S2732494X25000105sup001.pdf]

Supplemental Material for: Causal effects of empiric antibiotics on risk of Clostridioides difficile—a target trial emulation cohort study

Matthew A Pappas, MD, MPH, FHM; Shoshana J Herzig, MD; Andrew D Auerbach, MD, MPH; Abhishek Deshpande, MD, PhD; Eunice Blanchard, MS; Michael B Rothberg, MD, MPH

1. Description of target trial emulation design approach 3
2. Simplified directed acyclic graph (DAG) 4
3. Antibiotic daily dose-equivalents 5
4. Further detail on models predicting receipt of each antibiotic 6
5. Discrimination and calibration of models predicting receipt of each antibiotic 10
6. Balance after weighting 18
7. Post-hoc analysis comparing azithromycin against no antibiotics 33
8. Results of sensitivity analyses with inclusion/exclusion at 24 or 72 hours after admission, rather than 48 35

1. Description of target trial emulation design approach

We used a target trial emulation framework to guide our study design and analytic choices. Specifically, our causal inference observational analysis is meant to emulate a series of hypothetical unblinded randomized trials where patients were randomly assigned to receive one daily dose-equivalent of each of a series of antibiotics, described as follows:

Eligibility criteria:	Patients hospitalized at an HCA Healthcare inpatient facility for care of COVID-19 before widespread availability of vaccines (defined as before February 11, 2021), who are on day 3 or more of hospitalization, have not been previously diagnosed with CDI during the admission, and are not planned for discharge today. (Note: Our target trial would exclude patients *planned* for discharge on each calendar day; we excluded patients who actually left the hospital on each calendar day, which would not strictly be known at the time of treatment assignment. To the extent that discharges were unplanned, or that patients who were expected to leave remained hospitalized, this assumption could be a source of error.) We also studied, as sensitivity analyses, eligibility criteria which would be identical other than starting on day 2 or day 4 of hospitalization rather than day 3.
Treatment strategies:	Administration of one daily dose-equivalent of azithromycin or placebo (trial 1), ceftriaxone or placebo (trial 2), cefepime or placebo (trial 3), piperacillin/tazobactam or placebo (trial 4), meropenem or placebo (trial 5), levofloxacin or placebo (trial 6), or parenteral vancomycin or placebo (trial 7).
Assignment:	Neither participants nor physicians would be blinded to assignment for each antibiotic randomization.
Follow-up:	For each participant, follow-up would begin the calendar day after assignment and continue until hospital discharge.
Outcomes:	Diagnosis of CDI, defined as the combination of use of the ICD-10 code A04.72 and administration of one or more doses of metronidazole (oral or parenteral), oral vancomycin, or fidaxomicin.
Causal contrasts:	Intention-to-treat effect for one daily dose-equivalent versus placebo. Patients could be randomized on multiple calendar days, provided other eligibility criteria above are fulfilled.

2. Simplified directed acyclic graph (DAG)

We hypothesized that antimicrobial agents causally increase the risk of hospital-onset CDI, with the following DAG, where A represents the exposure (a daily dose-equivalent of each antibiotic), Y represents the outcome (hospital-onset CDI), and L represents measured confounders. Our treatment models used variables likely to be associated with receipt of empiric antimicrobials, including vital signs. Our outcome model used variables associated with hospital-onset CDI, including admission from a nursing facility.

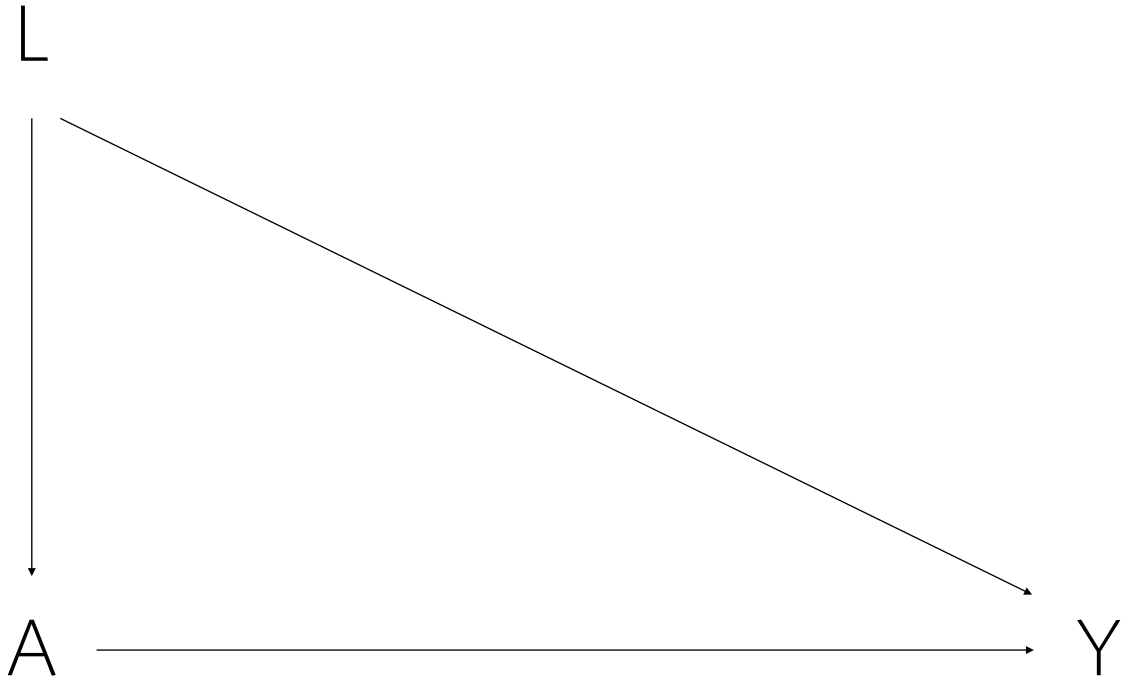

In building our models for receipt of each antibiotic (L->A), we focused on identifying confounders that would plausibly be used in antibiotic selection (such as vital signs), while also including key predictors of CDI (such as age and admission from a nursing facility). In building our outcome models (L->Y), data were more limited and we focused only on predictors of CDI.

3. Antibiotic daily dose-equivalents

We considered the following doses to represent one day of treatment:

- Azithromycin: 250 mg
- Ceftriaxone: 1 gram
- Cefepime: 1 gram
- Piperacillin/tazobactam: 13.5 grams (corresponding to either 3.375 grams Q6H or 4.5 grams Q8H)
- Meropenem: 3 grams (1 gram Q8H)
- Levofloxacin: 750 mg
- Vancomycin: 15 mg/kg of body weight

We analyzed by patient-day, such that on each day of analysis exposure was dichotomous, as greater than or equal to each of the above doses.

Because many hospital pharmacies dispense IV vancomycin in aliquots for oral administration, we parsed the administration instructions to ensure that we captured exposure to parenteral vancomycin alone, without also capturing doses of oral vancomycin. We used doses and administration instructions to identify when IV formulations of vancomycin were given orally.

4. Further detail on models predicting receipt of each antibiotic

Because administration of an antibiotic and antibiotic selection depend on physician factors and local practice patterns, we used multilevel (mixed effect) logistic regression models for each antibiotic. These models used variables at the level of the patient-day (that day's temperature, systolic blood pressure, heart rate, respiratory rate, and white blood cell count, whether the patient received the antibiotic in question on the previous calendar day, length of stay to date), at the level of the patient/hospitalization (age in years, biological sex, and whether the patient was admitted from a nursing facility), and the hospital ID. In the event of model nonconvergence despite any of our attempted numerical optimization algorithms or other numerical approaches, we used two-level models with patient-days clustered within patient/hospitalization with hospital ID as an indicator variable at the level of the hospitalization.

In each case, we used multiple imputation with chained equations (MICE) to minimize error from missing data. Of 922,187 patient-days, proportions of missing data were as follows:

**Variable:**	**Percent of observations missing:**
Heart rate	0.3%
Temperature	0.8%
Respiratory rate	0.8%
Systolic blood pressure	1.1%
WBC count	27.0%

We imputed each of those variables using linear regression models. Other variables were either considered documented by exception and therefore never missing (e.g., admission from a nursing facility) or had trivial missingness (e.g., age, sex). Following the rule of thumb that the number of imputations should at least exceed the highest percentage of missing data, we used 60 imputations. We verified that MICE did not introduce Monte Carlo error based on the White, Royston, and Wood guidelines. The fraction of missing information (FMI, which is the proportion of total sampling variance that is due to missing data) for variables included in each prediction model are shown below.

Azithromycin:

**Variable:**	**Fraction of missing information:**
Heart rate, restricted cubic spline 1	0.4%
Heart rate, restricted cubic spline 2	0.3%
Heart rate, restricted cubic spline 3	0.2%
Heart rate, restricted cubic spline 4	0.2%
Temperature, restricted cubic spline 1	0.8%
Temperature, restricted cubic spline 2	0.7%
Temperature, restricted cubic spline 3	0.8%
Temperature, restricted cubic spline 4	0.8%
Respiratory rate, restricted cubic spline 1	0.6%
Respiratory rate, restricted cubic spline 2	0.4%
Respiratory rate, restricted cubic spline 3	0.3%
Respiratory rate, restricted cubic spline 4	0.2%
Systolic blood pressure, restricted cubic spline 1	0.7%
Systolic blood pressure, restricted cubic spline 2	0.5%
Systolic blood pressure, restricted cubic spline 3	0.5%
Systolic blood pressure, restricted cubic spline 4	0.5%
WBC count, restricted cubic spline 1	44.2%

WBC count, restricted cubic spline 2	32.6%
WBC count, restricted cubic spline 3	26.5%
WBC count, restricted cubic spline 4	23.1%

Ceftriaxone:

**Variable:**	**Fraction of missing information:**
Heart rate, restricted cubic spline 1	0.6%
Heart rate, restricted cubic spline 2	0.3%
Heart rate, restricted cubic spline 3	0.2%
Heart rate, restricted cubic spline 4	0.2%
Temperature, restricted cubic spline 1	0.7%
Temperature, restricted cubic spline 2	0.6%
Temperature, restricted cubic spline 3	0.6%
Temperature, restricted cubic spline 4	0.6%
Respiratory rate, restricted cubic spline 1	6.8%
Respiratory rate, restricted cubic spline 2	4.6%
Respiratory rate, restricted cubic spline 3	3.5%
Respiratory rate, restricted cubic spline 4	2.8%
Systolic blood pressure, restricted cubic spline 1	1.7%
Systolic blood pressure, restricted cubic spline 2	1.4%
Systolic blood pressure, restricted cubic spline 3	1.1%
Systolic blood pressure, restricted cubic spline 4	0.9%
WBC count, restricted cubic spline 1	52.2%
WBC count, restricted cubic spline 2	39.5%
WBC count, restricted cubic spline 3	31.3%
WBC count, restricted cubic spline 4	25.2%

Cefepime:

**Variable:**	**Fraction of missing information:**
Heart rate, restricted cubic spline 1	1.4%
Heart rate, restricted cubic spline 2	1.2%
Heart rate, restricted cubic spline 3	1.0%
Heart rate, restricted cubic spline 4	0.9%
Temperature, restricted cubic spline 1	0.7%
Temperature, restricted cubic spline 2	1.5%
Temperature, restricted cubic spline 3	1.5%
Temperature, restricted cubic spline 4	1.5%
Respiratory rate, restricted cubic spline 1	7.1%
Respiratory rate, restricted cubic spline 2	4.0%
Respiratory rate, restricted cubic spline 3	3.0%
Respiratory rate, restricted cubic spline 4	2.5%
Systolic blood pressure, restricted cubic spline 1	4.9%
Systolic blood pressure, restricted cubic spline 2	3.5%
Systolic blood pressure, restricted cubic spline 3	2.9%
Systolic blood pressure, restricted cubic spline 4	2.5%
WBC count, restricted cubic spline 1	35.2%
WBC count, restricted cubic spline 2	25.8%
WBC count, restricted cubic spline 3	20.2%
WBC count, restricted cubic spline 4	16.2%

Piperacillin/tazobactam:

Variable:	Fraction of missing information:
Heart rate, restricted cubic spline 1	1.4%
Heart rate, restricted cubic spline 2	1.3%
Heart rate, restricted cubic spline 3	1.2%
Heart rate, restricted cubic spline 4	1.0%
Temperature, restricted cubic spline 1	0.5%
Temperature, restricted cubic spline 2	1.6%
Temperature, restricted cubic spline 3	1.7%
Temperature, restricted cubic spline 4	1.7%
Respiratory rate, restricted cubic spline 1	8.1%
Respiratory rate, restricted cubic spline 2	5.1%
Respiratory rate, restricted cubic spline 3	4.0%
Respiratory rate, restricted cubic spline 4	3.3%
Systolic blood pressure, restricted cubic spline 1	4.9%
Systolic blood pressure, restricted cubic spline 2	4.2%
Systolic blood pressure, restricted cubic spline 3	3.6%
Systolic blood pressure, restricted cubic spline 4	2.9%
WBC count, restricted cubic spline 1	49.0%
WBC count, restricted cubic spline 2	40.6%
WBC count, restricted cubic spline 3	33.5%
WBC count, restricted cubic spline 4	26.3%

Meropenem:

Variable:	Fraction of missing information:
Heart rate, restricted cubic spline 1	0.9%
Heart rate, restricted cubic spline 2	0.8%
Heart rate, restricted cubic spline 3	0.8%
Heart rate, restricted cubic spline 4	0.7%
Temperature, restricted cubic spline 1	0.4%
Temperature, restricted cubic spline 2	1.0%
Temperature, restricted cubic spline 3	1.1%
Temperature, restricted cubic spline 4	1.1%
Respiratory rate, restricted cubic spline 1	7.0%
Respiratory rate, restricted cubic spline 2	4.0%
Respiratory rate, restricted cubic spline 3	3.0%
Respiratory rate, restricted cubic spline 4	2.4%
Systolic blood pressure, restricted cubic spline 1	6.6%
Systolic blood pressure, restricted cubic spline 2	5.4%
Systolic blood pressure, restricted cubic spline 3	4.7%
Systolic blood pressure, restricted cubic spline 4	4.0%
WBC count, restricted cubic spline 1	36.6%
WBC count, restricted cubic spline 2	28.1%
WBC count, restricted cubic spline 3	21.6%
WBC count, restricted cubic spline 4	16.6%

Levofloxacin:

Variable:	Fraction of missing information:
Heart rate, restricted cubic spline 1	0.3%
Heart rate, restricted cubic spline 2	0.3%
Heart rate, restricted cubic spline 3	0.3%

Heart rate, restricted cubic spline 4	0.2%
Temperature, restricted cubic spline 1	0.4%
Temperature, restricted cubic spline 2	0.4%
Temperature, restricted cubic spline 3	0.4%
Temperature, restricted cubic spline 4	0.4%
Respiratory rate, restricted cubic spline 1	6.2%
Respiratory rate, restricted cubic spline 2	3.7%
Respiratory rate, restricted cubic spline 3	2.7%
Respiratory rate, restricted cubic spline 4	2.1%
Systolic blood pressure, restricted cubic spline 1	2.2%
Systolic blood pressure, restricted cubic spline 2	1.6%
Systolic blood pressure, restricted cubic spline 3	1.3%
Systolic blood pressure, restricted cubic spline 4	1.0%
WBC count, restricted cubic spline 1	40.0%
WBC count, restricted cubic spline 2	34.5%
WBC count, restricted cubic spline 3	30.8%
WBC count, restricted cubic spline 4	26.8%

Vancomycin:

**Variable:**	**Fraction of missing information:**
Heart rate, restricted cubic spline 1	0.4%
Heart rate, restricted cubic spline 2	0.3%
Heart rate, restricted cubic spline 3	0.3%
Heart rate, restricted cubic spline 4	0.3%
Temperature, restricted cubic spline 1	0.2%
Temperature, restricted cubic spline 2	0.7%
Temperature, restricted cubic spline 3	0.7%
Temperature, restricted cubic spline 4	0.8%
Respiratory rate, restricted cubic spline 1	4.7%
Respiratory rate, restricted cubic spline 2	3.0%
Respiratory rate, restricted cubic spline 3	2.4%
Respiratory rate, restricted cubic spline 4	2.1%
Systolic blood pressure, restricted cubic spline 1	2.4%
Systolic blood pressure, restricted cubic spline 2	2.5%
Systolic blood pressure, restricted cubic spline 3	2.4%
Systolic blood pressure, restricted cubic spline 4	2.2%
WBC count, restricted cubic spline 1	42.0%
WBC count, restricted cubic spline 2	33.1%
WBC count, restricted cubic spline 3	26.1%
WBC count, restricted cubic spline 4	19.9%

5. Discrimination and calibration of models predicting receipt of each antibiotic

Assessing discrimination and calibration in multiply-imputed multilevel models can be challenging, and universally accepted approaches are lacking. For each antibiotic, we assessed discrimination and calibration using simplified versions without mixed effects/clustering. The discrimination and calibration measures below were calculated from single-level logistic regression models including all patient-day and patient-level variables, and with hospital ID as an indicator variable rather than as a mixed effect. Patient ID was not included in this simplified model structure. Calibration-in-the-large is also shown in the table below. Calibration plots for each antibiotic model show proportions of patient-days with receipt of the antibiotic (observed) plotted against model-predicted proportions (expected), binned into deciles.

	AUROC	Brier score	Mean prob. of outcome	Mean prob. of forecast
**Azithromycin**	0.93	0.09	0.26	0.26
**Ceftriaxone**	0.92	0.10	0.28	0.28
**Cefepime**	0.93	0.03	0.08	0.08
**Piperacillin/tazobactam**	0.93	0.03	0.06	0.06
**Meropenem**	0.94	0.01	0.02	0.02
**Levofloxacin**	0.86	0.01	0.01	0.01
**Vancomycin**	0.85	0.03	0.04	0.04

Azithromycin:

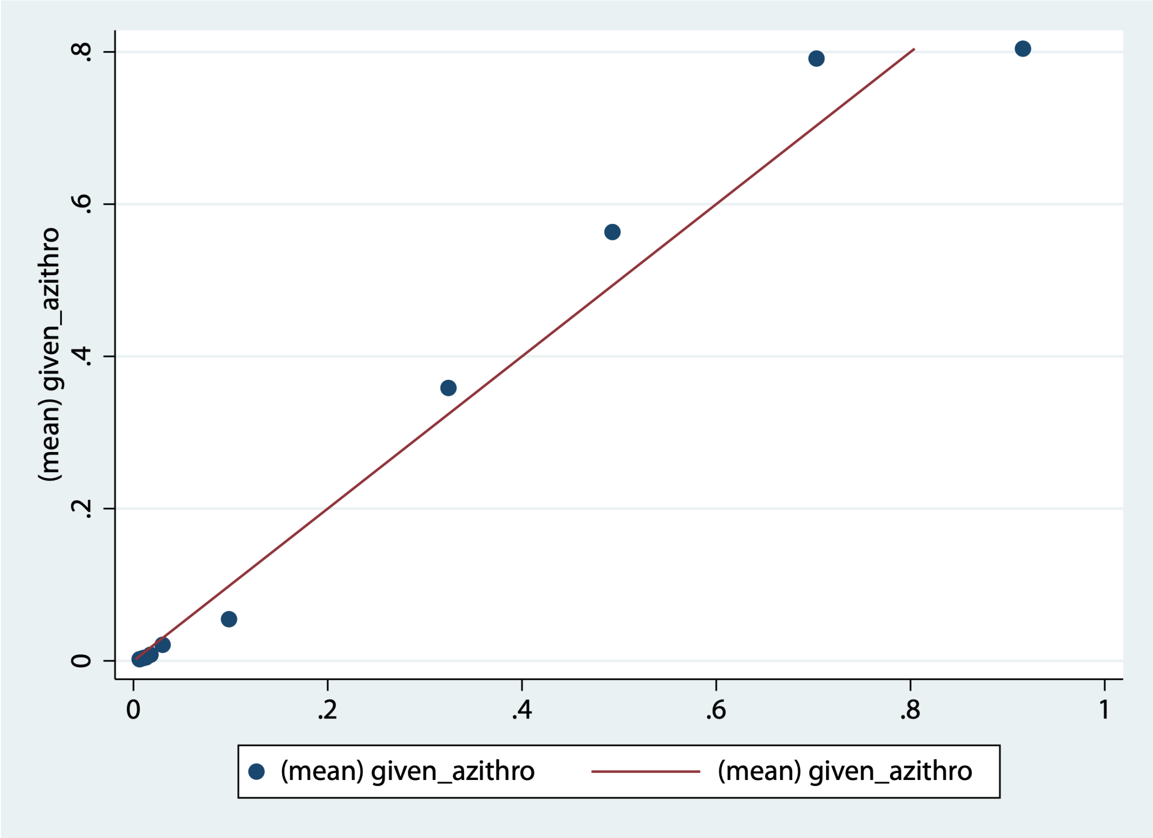

Ceftriaxone:

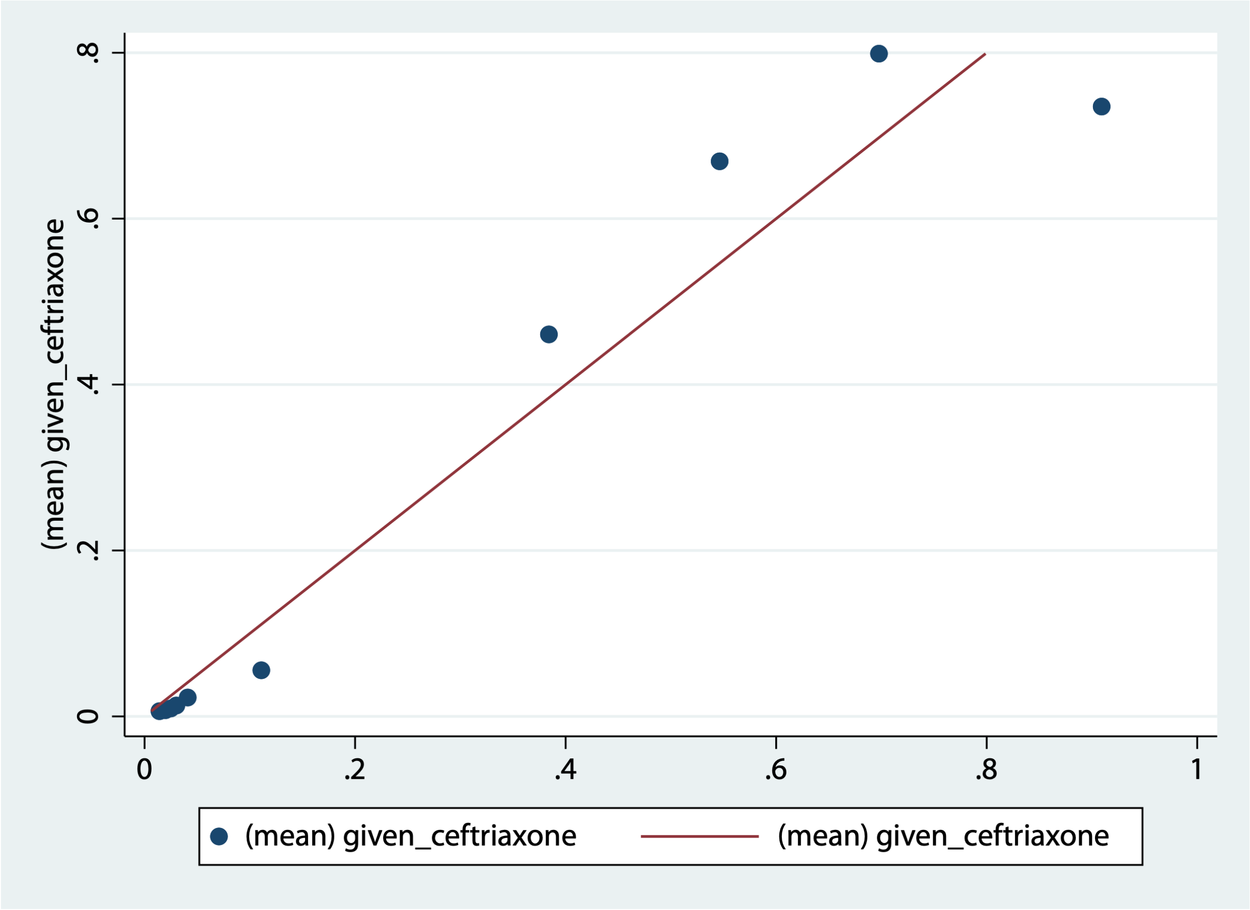

Cefepime:

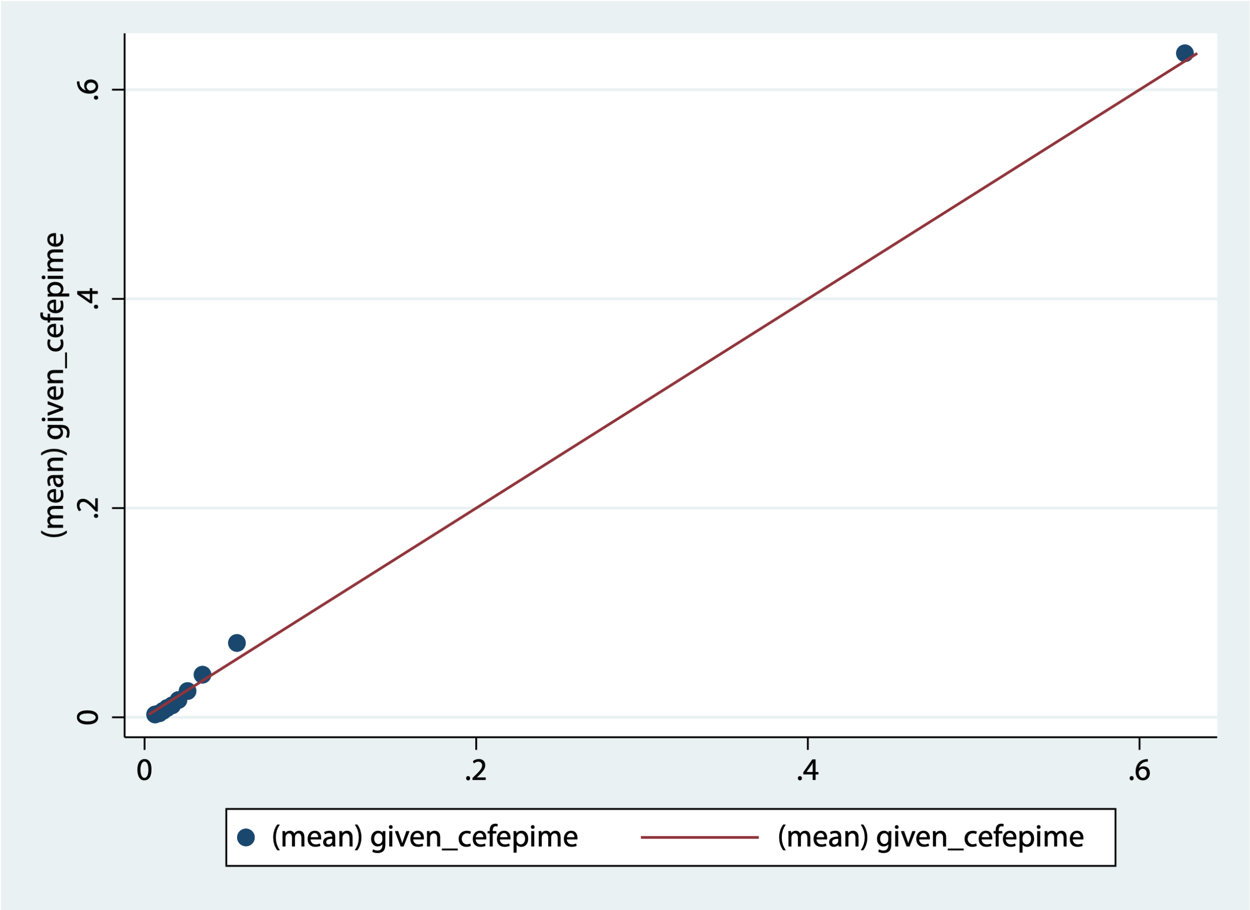

(mean) given_cefepime
0
.2
.4
.6
0
.2
.4
.6
(mean) given_cefepime
(mean) given_cefepime

Piperacillin/tazobactam

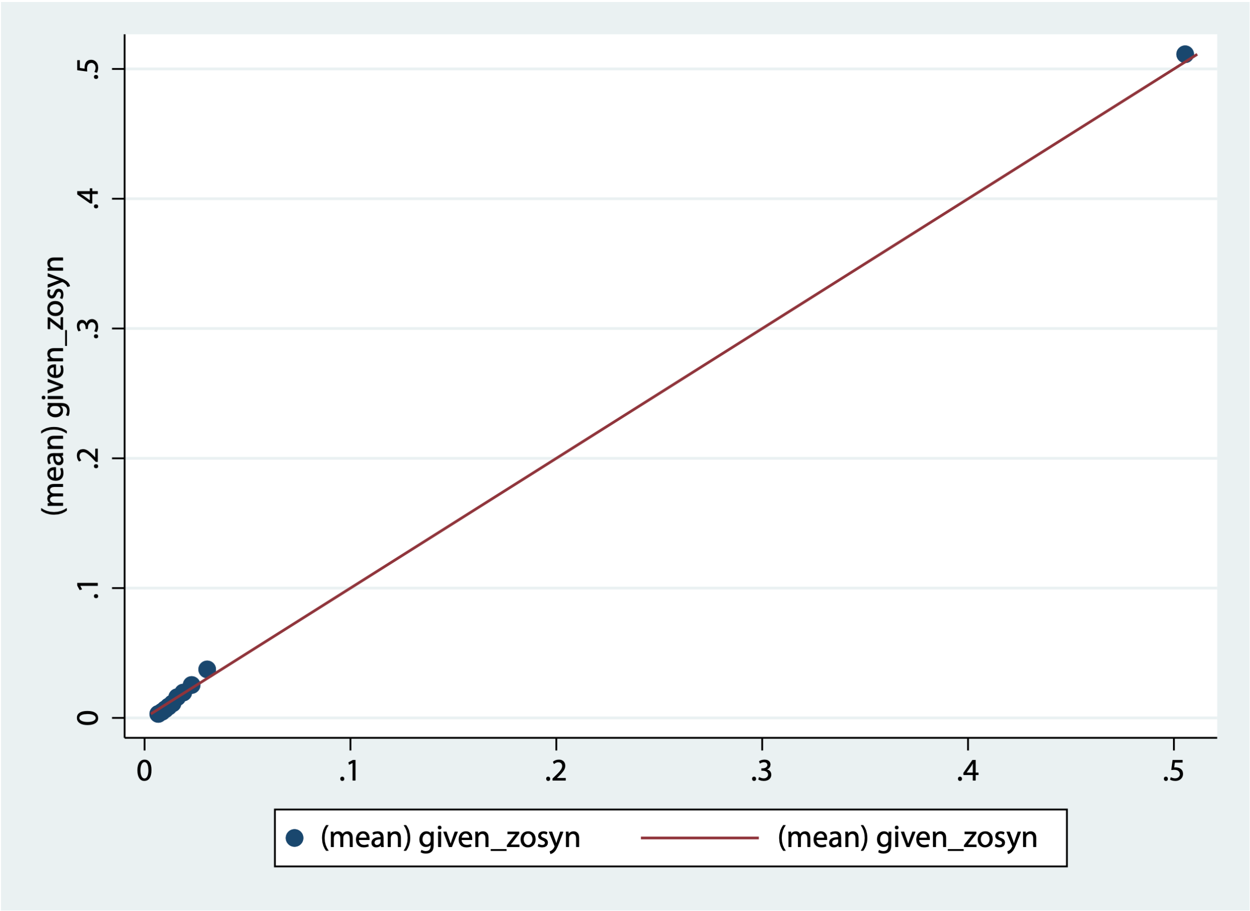

Meropenem

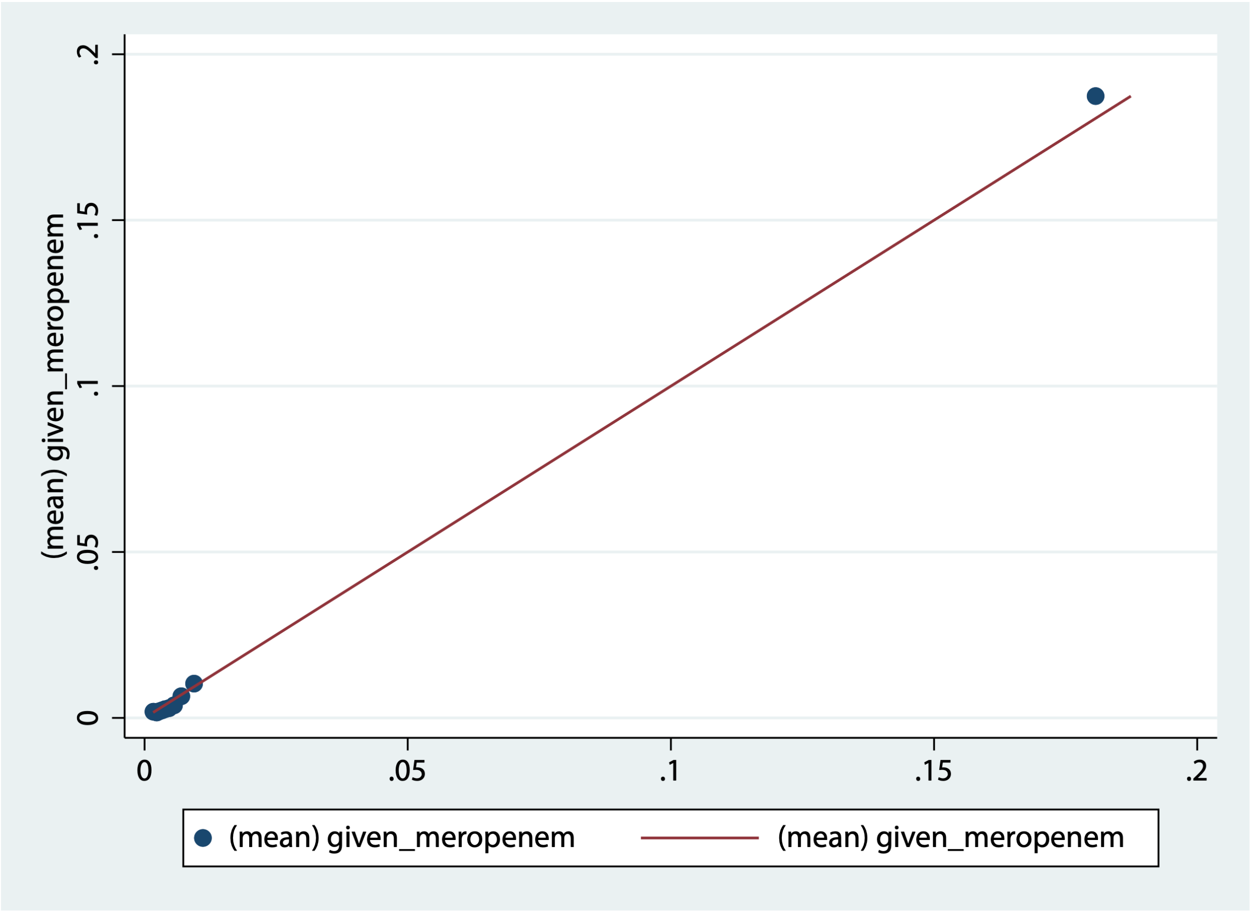

.2
.15
.1
.05
0
(mean) given_meropenem
0
.05
.1
.15
.2
(mean) given_meropenem
(mean) given_meropenem

Levofloxacin

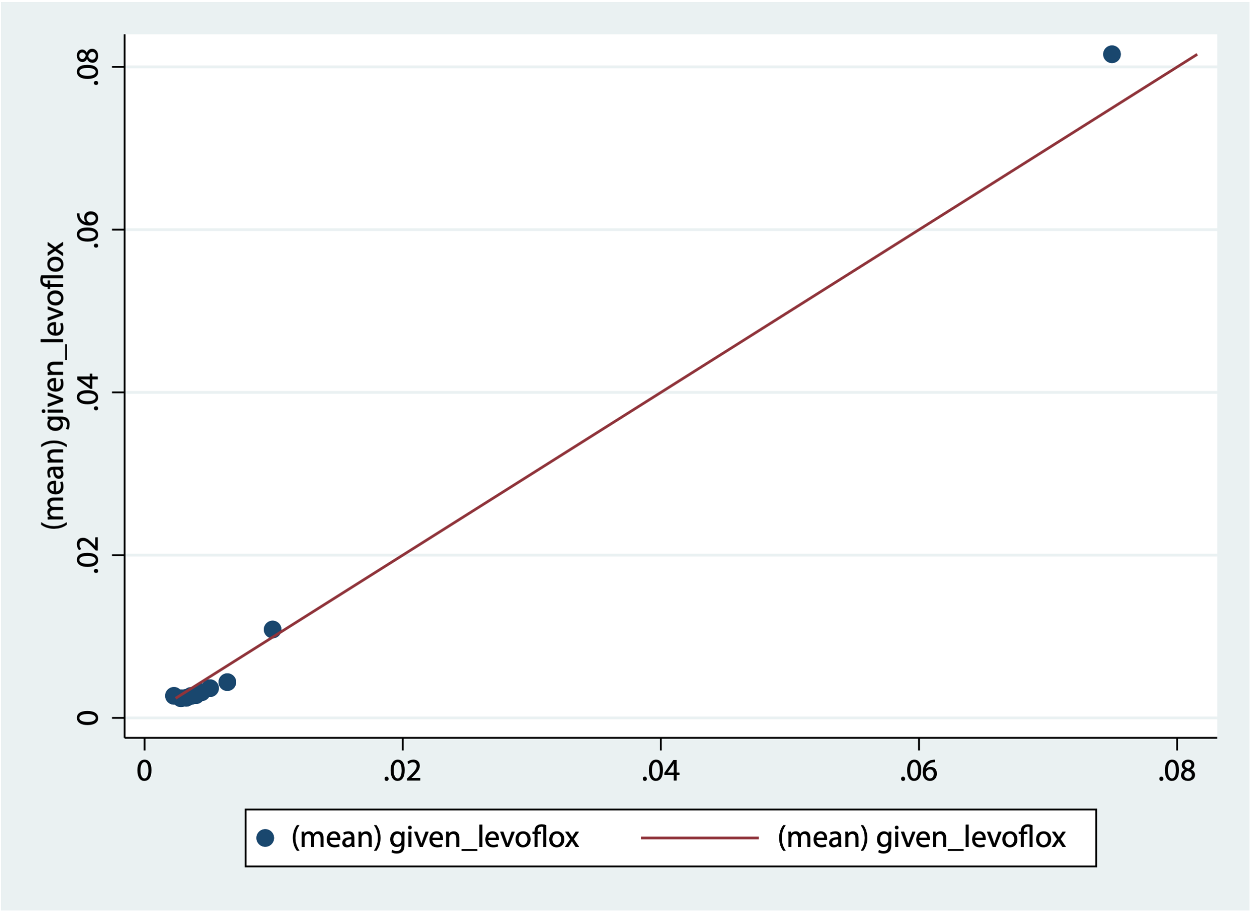

Parenteral vancomycin

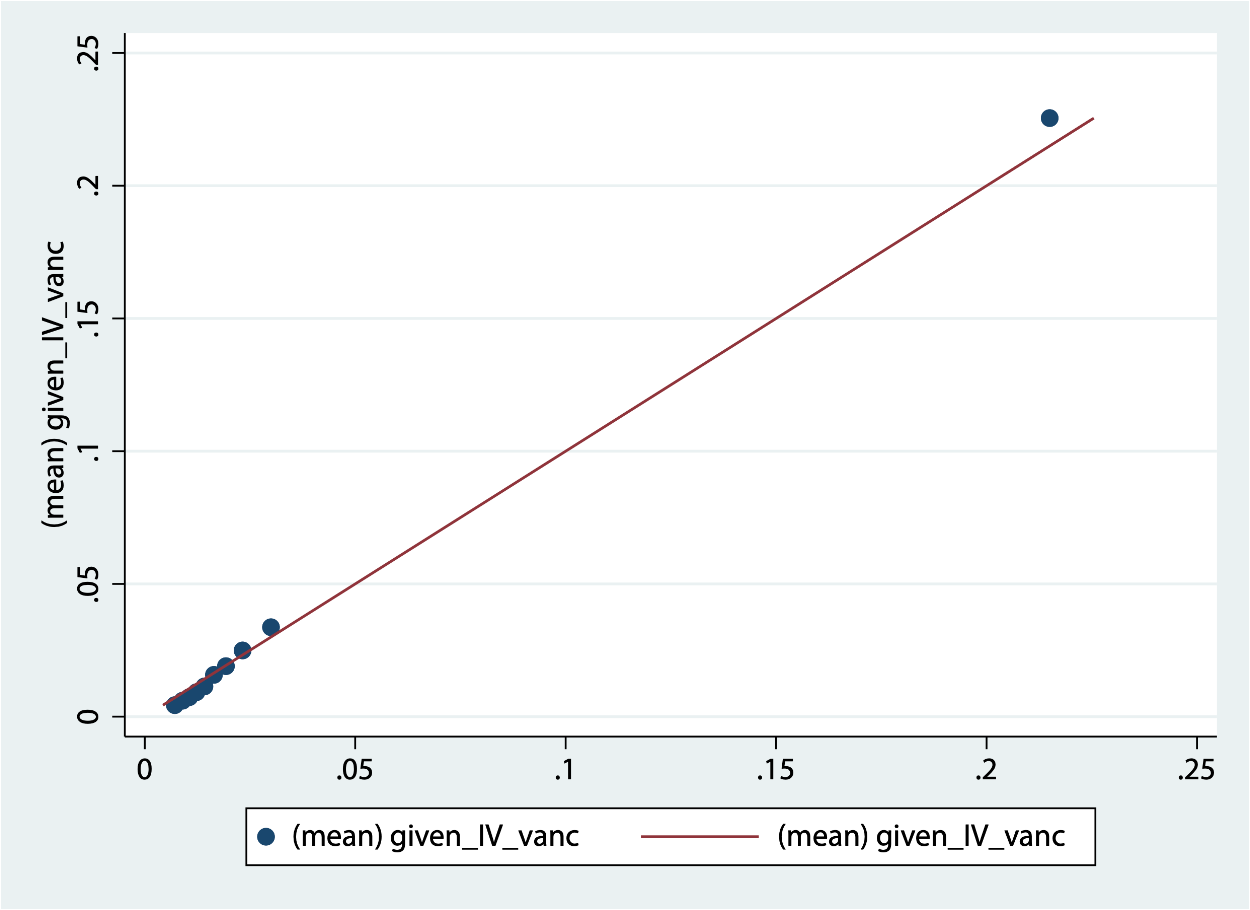

6. Balance after weighting

After matching by probability of receiving each antibiotic, we created density plots for visual bias inspection. For each antibiotic, we show the overlap plots and covariate balance after weighting below.

Azithromycin

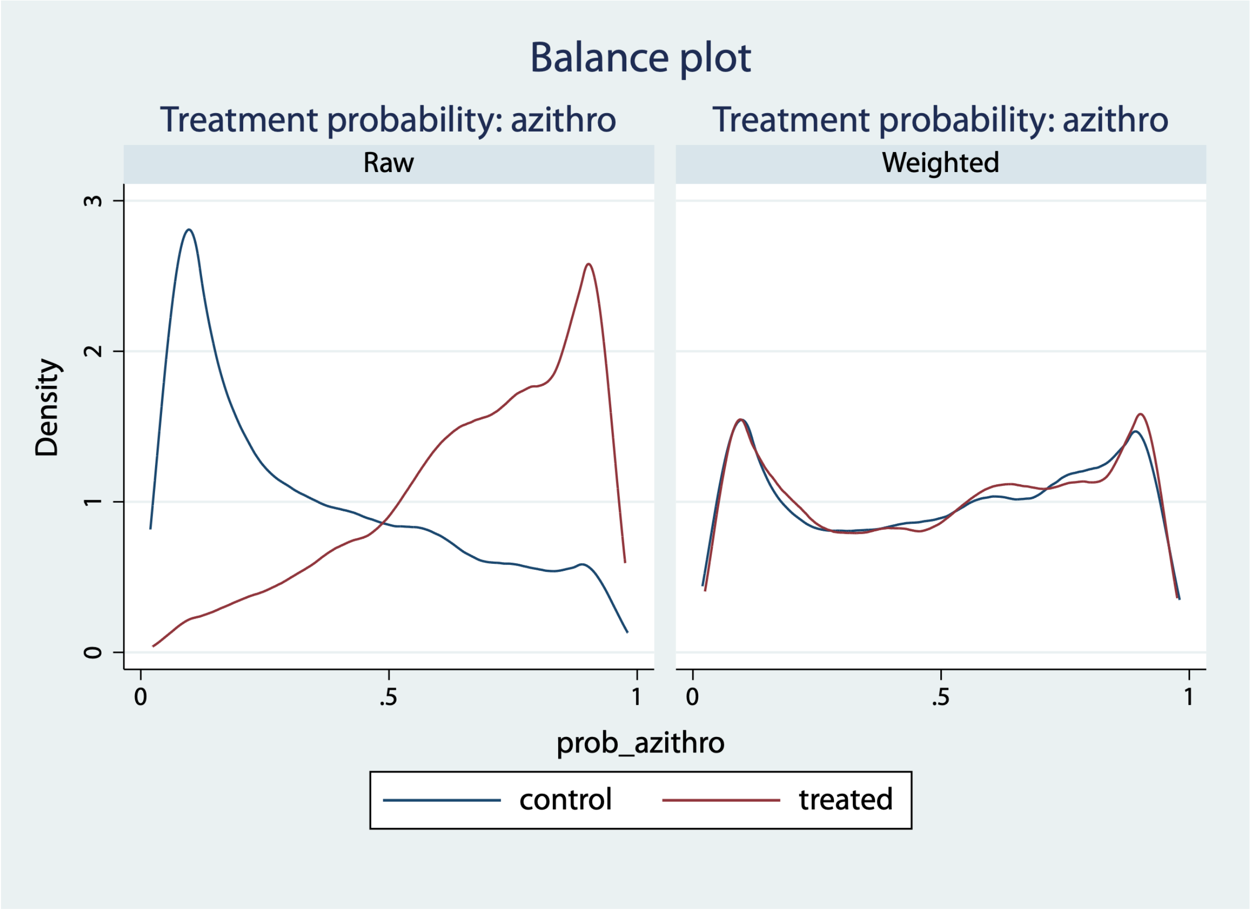

Covariate balance summary:

	Raw	Weighted
Number of obs =	52,627	52,627.0
Treated obs =	25,677	26,247.8
Control obs =	26,950	26,379.2

Ceftriaxone

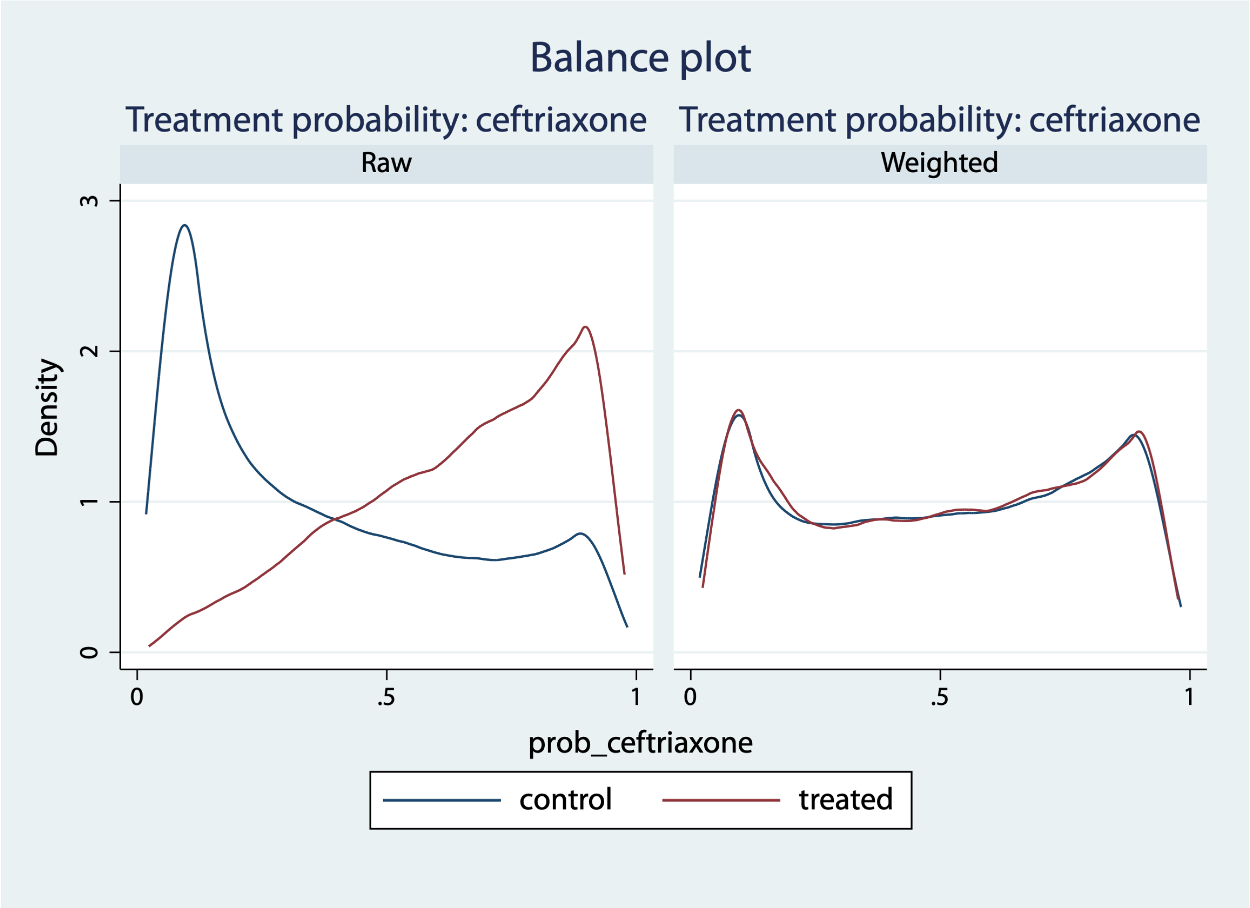

Covariate balance summary:

	Raw	Weighted
Number of obs =	55,789	55,789.0
Treated obs =	26,991	27,802.7
Control obs =	28,798	27,986.3

Cefepime

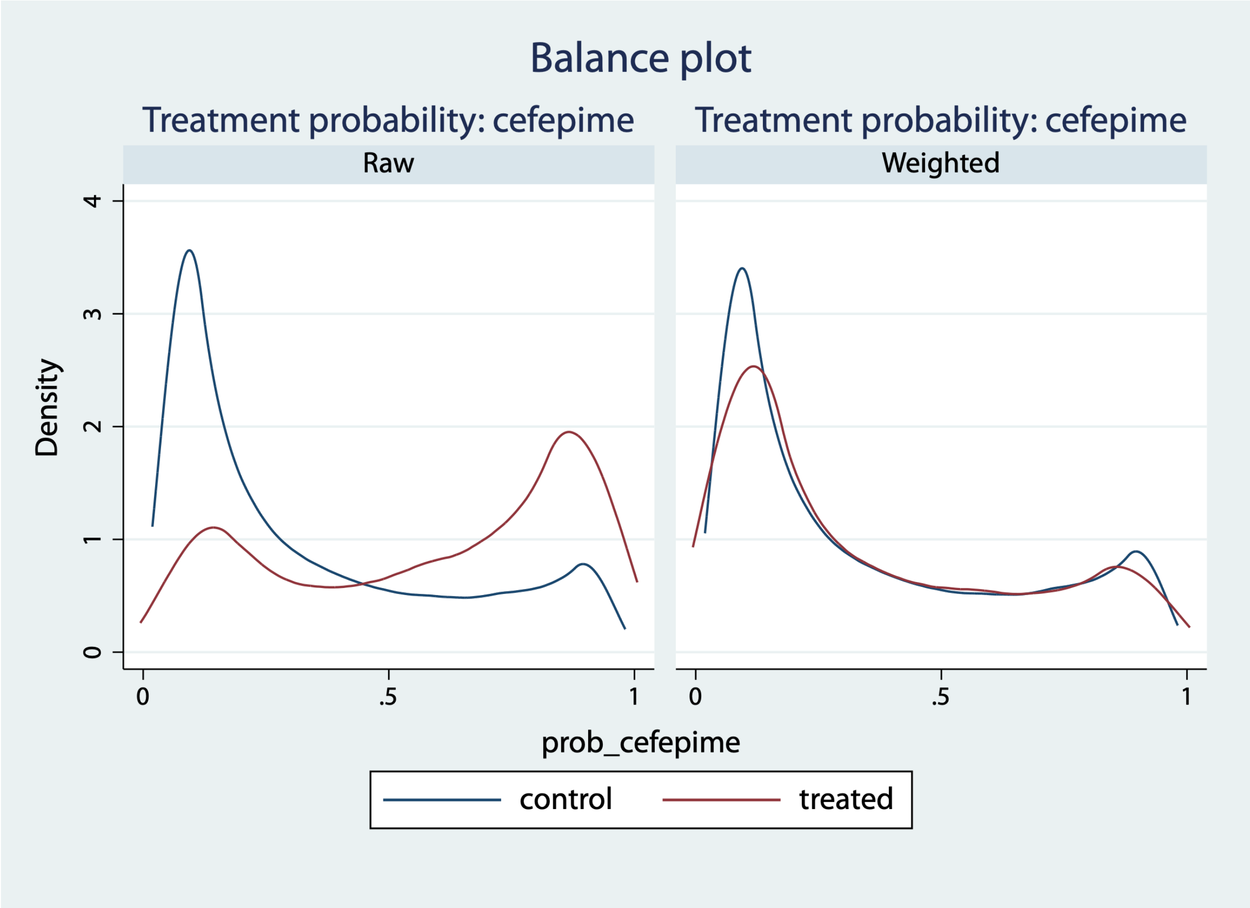

Covariate balance summary:

	Raw	Weighted
Number of obs =	38,003	38,003.0
Treated obs =	2,596	19,007.2
Control obs =	35,407	18,995.8

Piperacillin/tazobactam

This model did not converge in our initial effort. We therefore trimmed an additional 1% of observations, such that the causal effect model included patient-days between a 6% and 95% probability of administration.

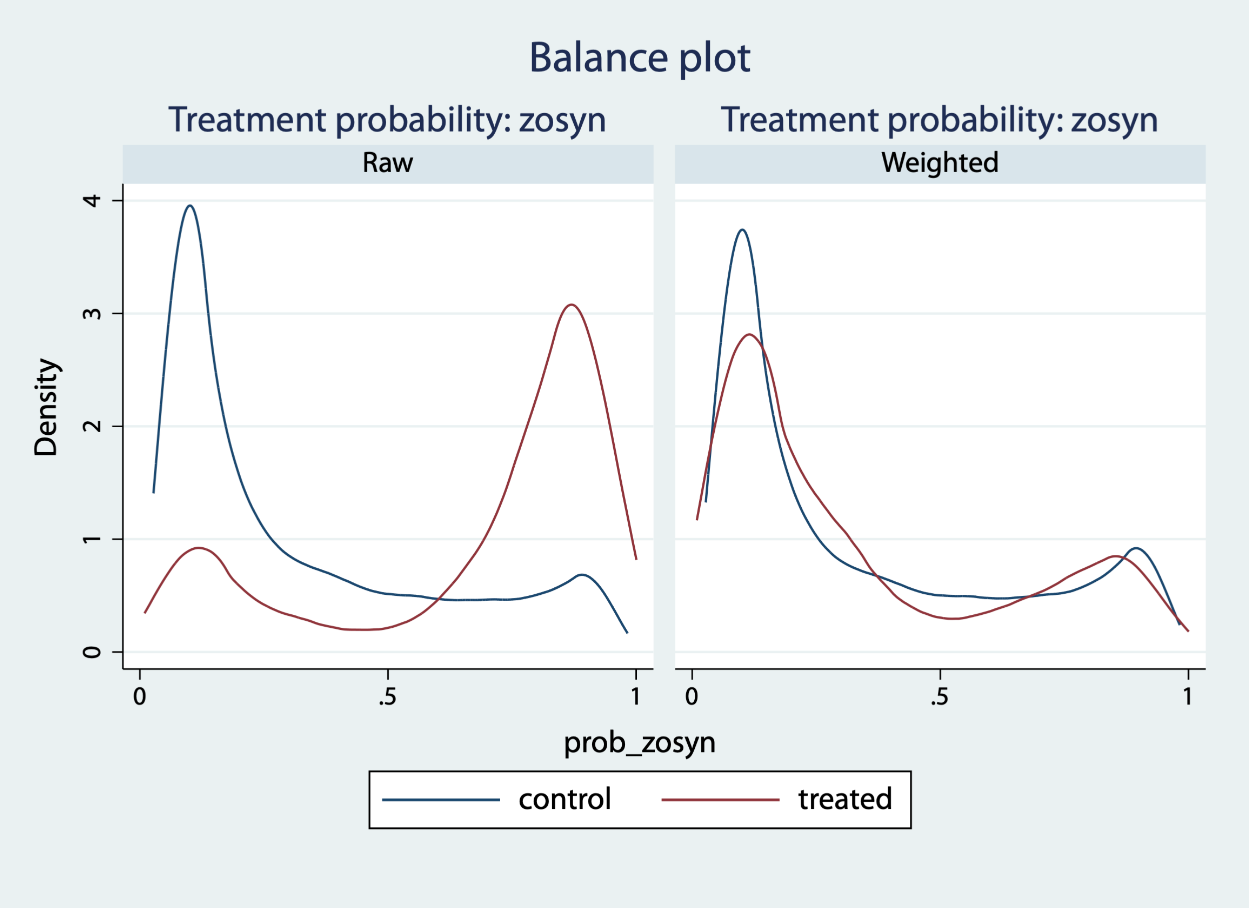

Covariate balance summary:

```
                              Raw      Weighted
-----------------------------------------------
Number of obs =            26,173      26,173.0
Treated obs   =             2,030      13,148.6
Control obs   =            24,143      13,024.4
-----------------------------------------------
```

Vancomycin

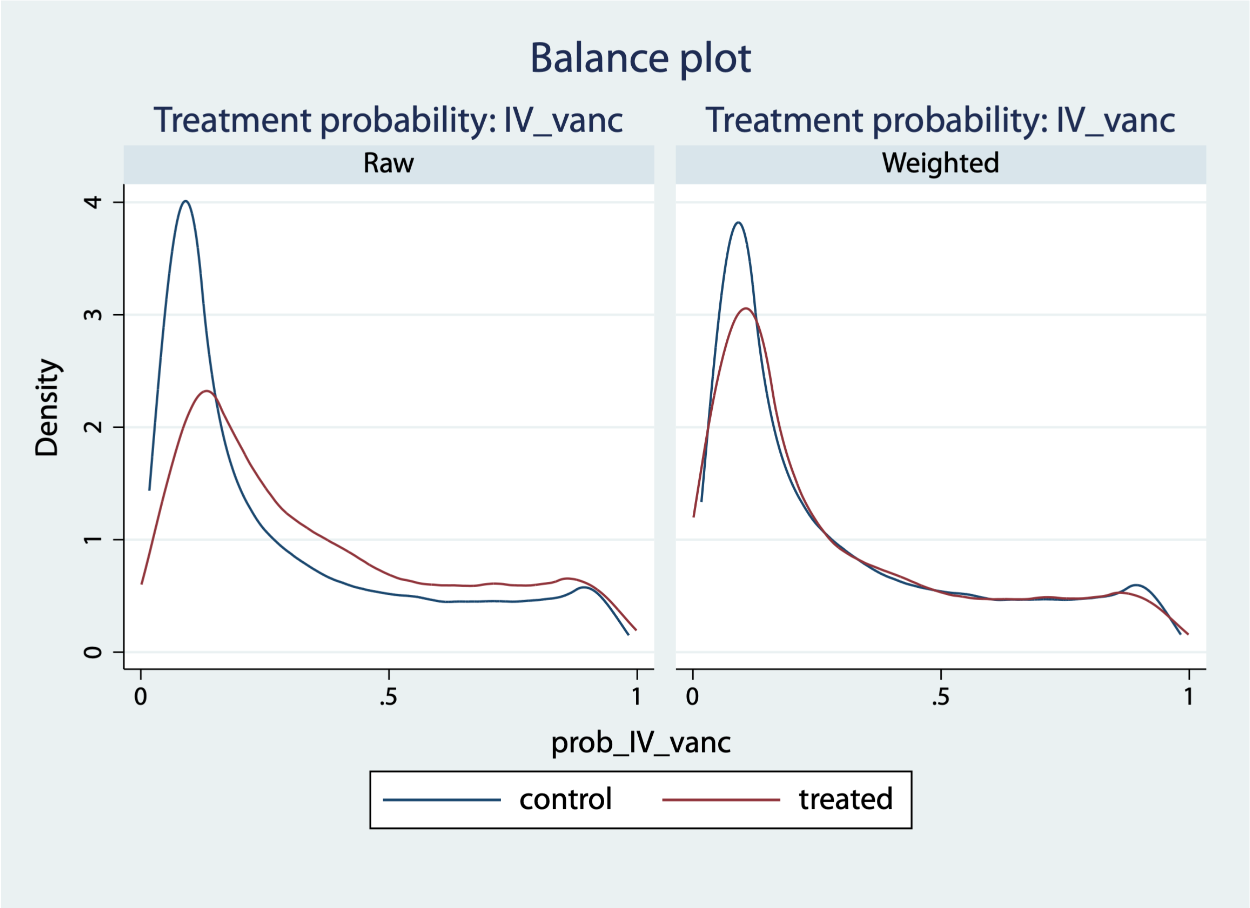

Covariate balance summary:

	Raw	Weighted
Number of obs =	24,406	24,406.0
Treated obs =	3,035	12,207.2
Control obs =	21,371	12,198.8

Below are overlap plots for our sensitivity analysis including patient-days after the first 24 hours in hospital, rather than after the first 48.

Azithromycin:

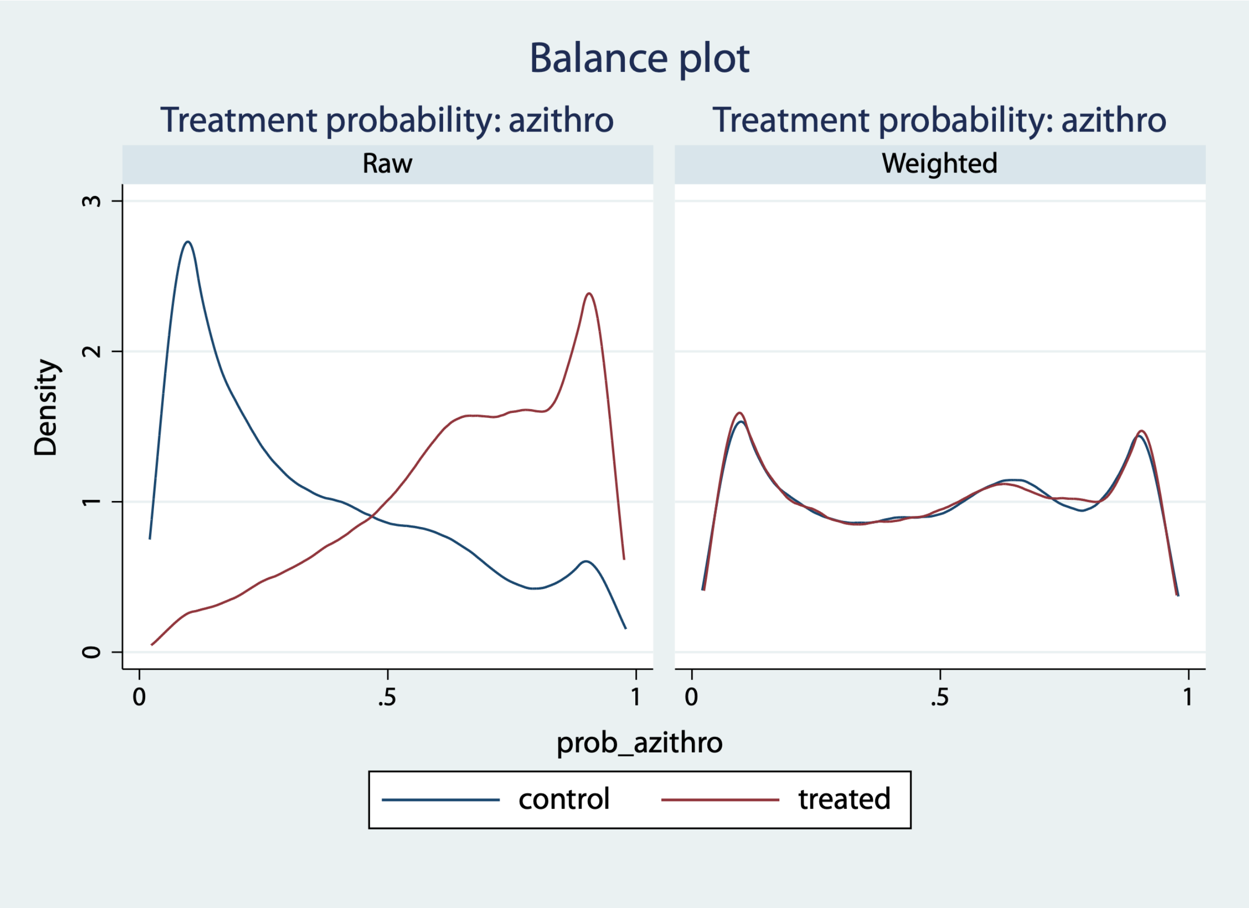

Covariate balance summary:

	Raw	Weighted
Number of obs =	60,189	60,189.0
Treated obs =	29,064	30,090.4
Control obs =	31,125	30,098.6

Ceftriaxone:

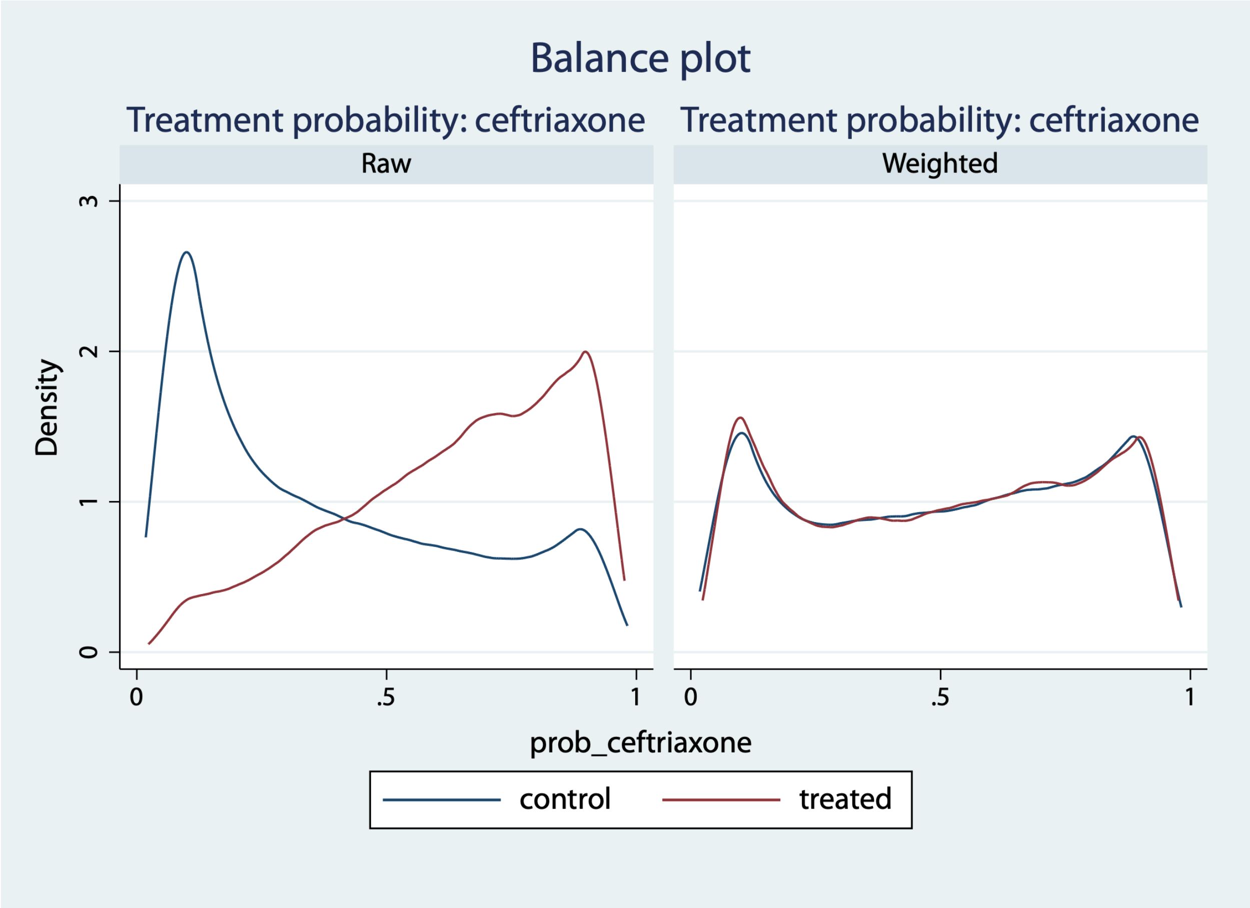

Covariate balance summary:

	Raw	Weighted
Number of obs =	59,005	59,005.0
Treated obs =	30,405	29,466.9
Control obs =	28,600	29,538.1

Cefepime:

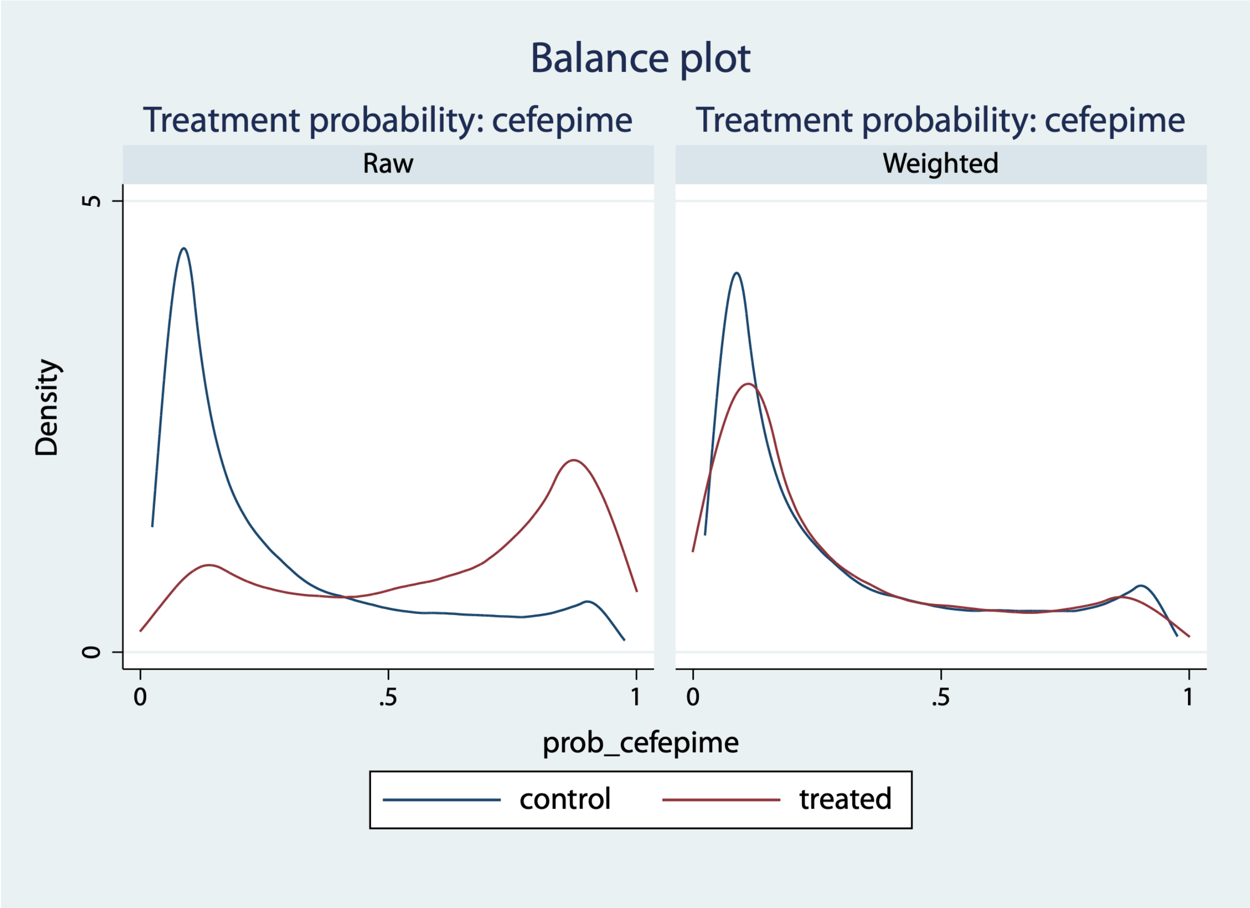

Covariate balance summary:

		Raw	Weighted
Number of obs	=	43,986	43,986.0
Treated obs	=	3,522	21,986.2
Control obs	=	40,464	21,999.8

Piperacillin/tazobactam:

This model did not initially converge. We trimmed observations in increments of 1% until achieving convergence. The causal effect model included patient-days between a 6% and 94% probability of administration.

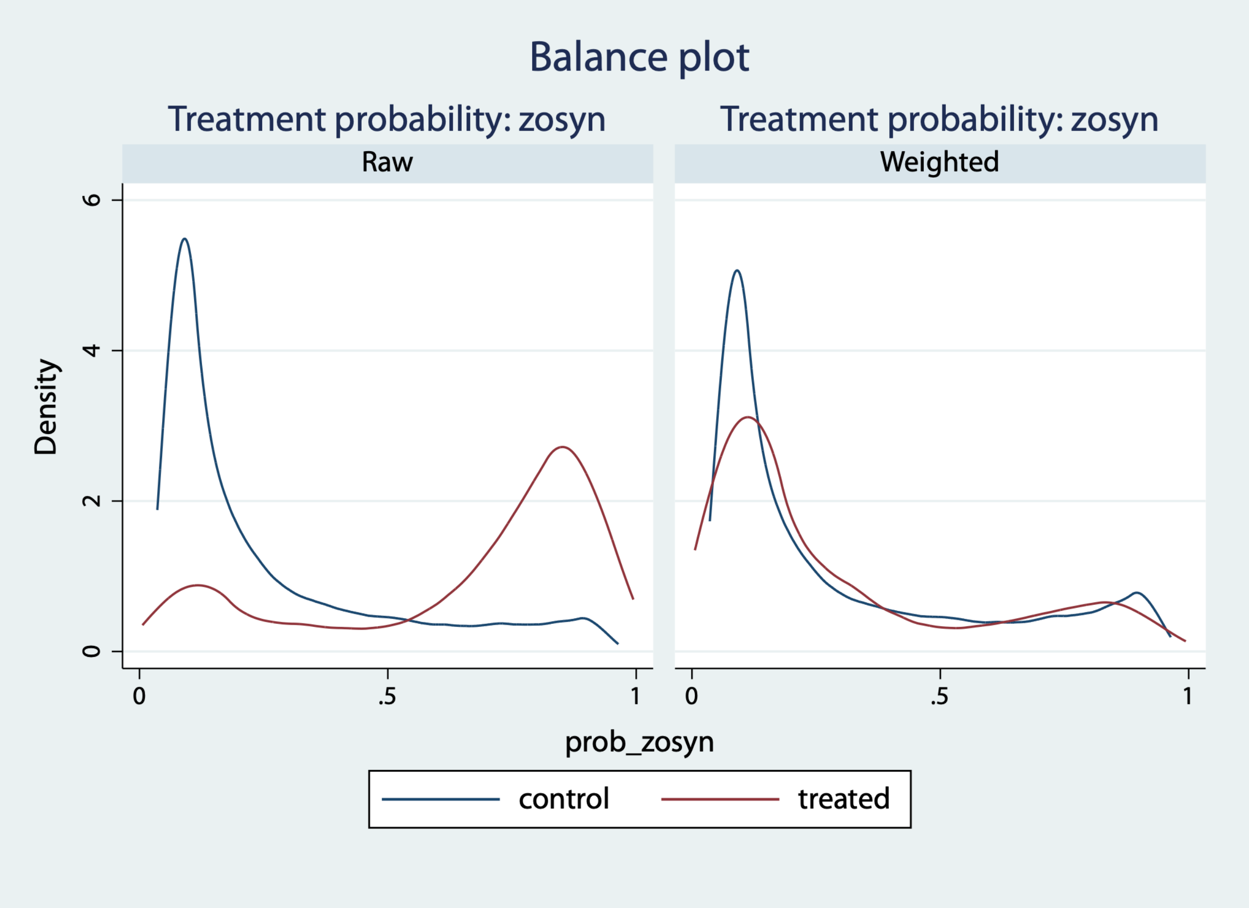

Covariate balance summary:

	Raw	Weighted
Number of obs =	23,419	23,419.0
Treated obs =	2,390	11,718.6
Control obs =	21,029	11,700.4

Vancomycin:

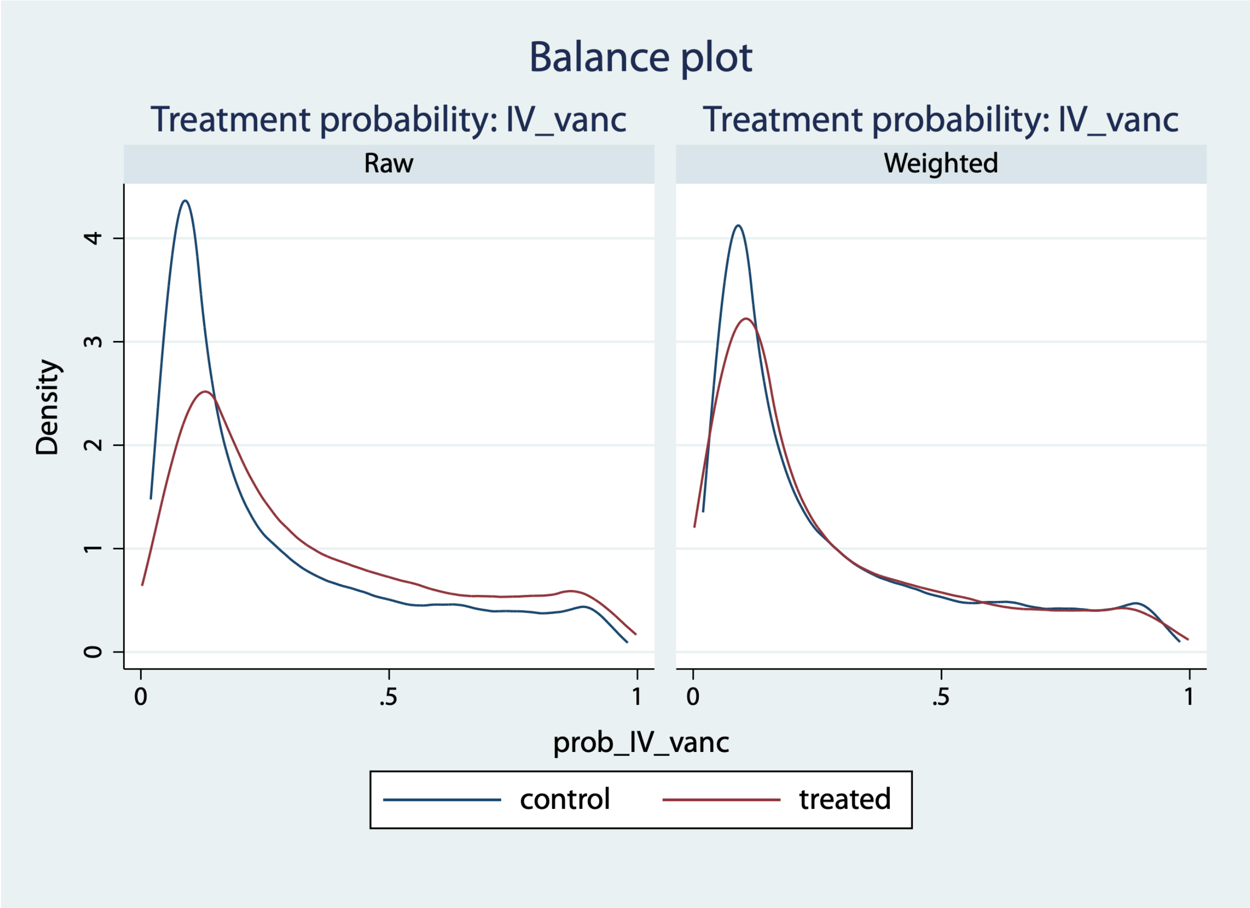

Covariate balance summary:

		Raw	Weighted
Number of obs	=	19,621	19,621.0
Treated obs	=	3,019	9,810.1
Control obs	=	16,602	9,810.9

Below are overlap plots for our sensitivity analysis including patient-days after the first 72 hours in hospital, rather than after the first 48.

Azithromycin:

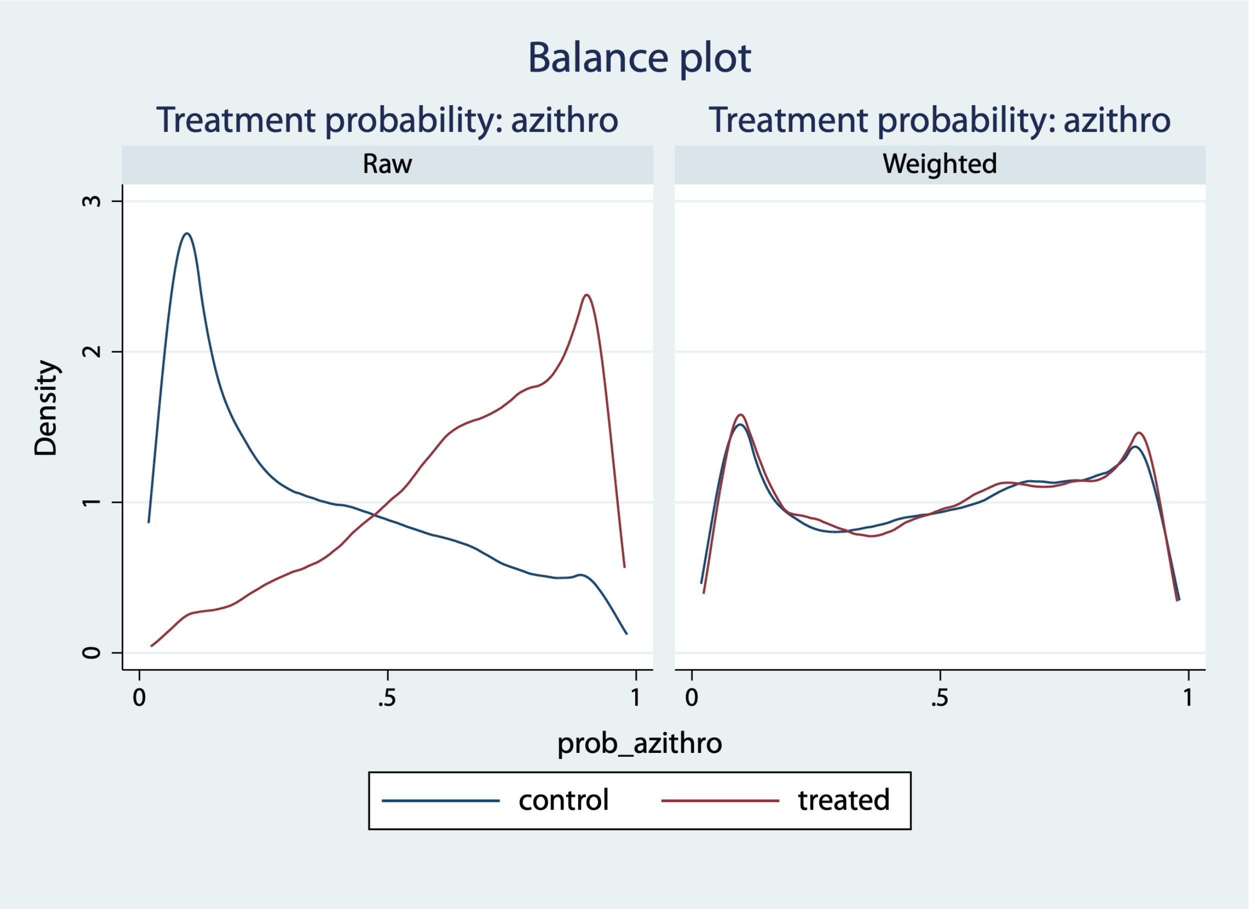

Covariate balance summary:

	Raw	Weighted
Number of obs =	44,207	44,207.0
Treated obs =	22,012	22,052.4
Control obs =	22,195	22,154.6

Ceftriaxone:

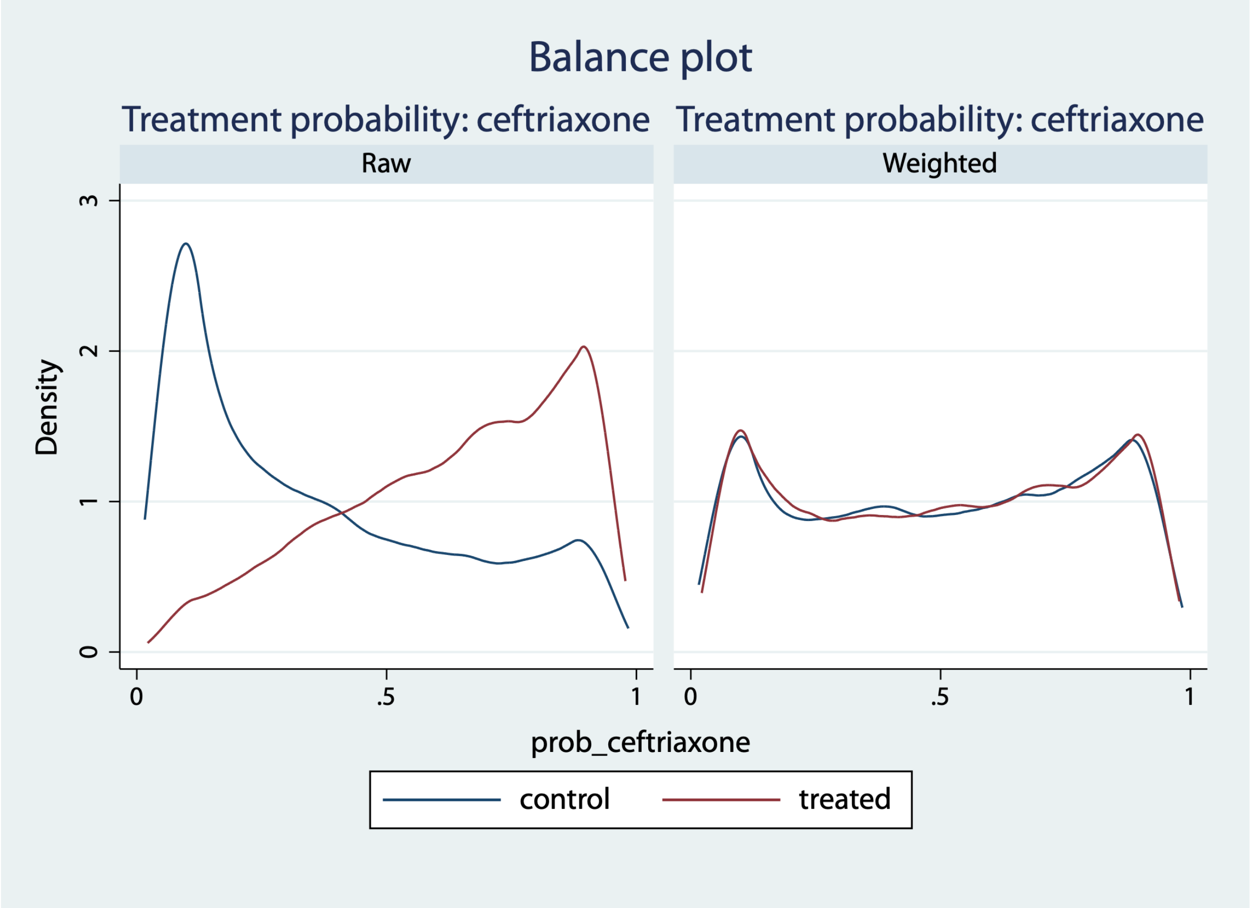

Covariate balance summary:

		Raw	Weighted
Number of obs	=	43,433	43,433.0
Treated obs	=	23,212	21,665.3
Control obs	=	20,221	21,767.7

Cefepime:

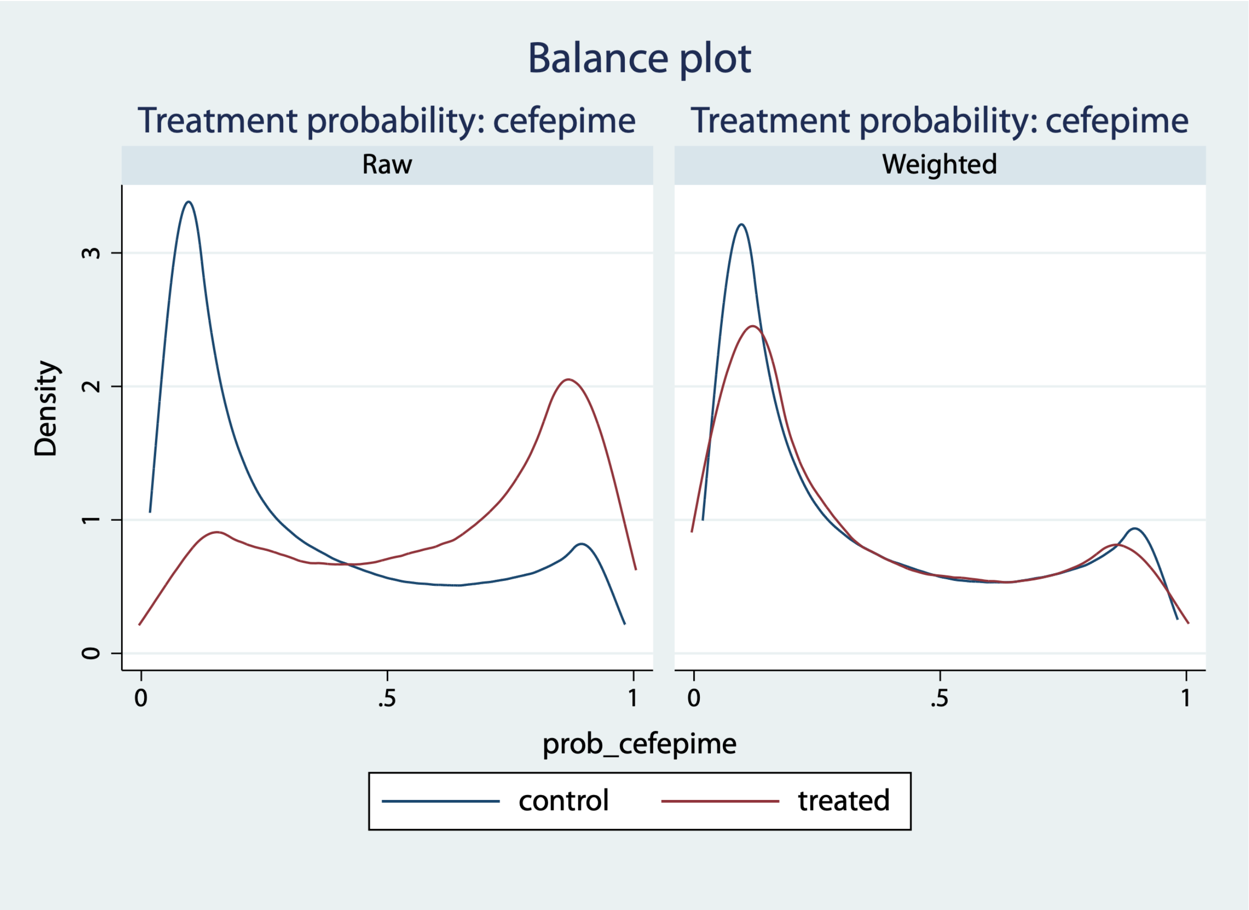

Covariate balance summary:

	Raw	Weighted
Number of obs =	33,734	33,734.0
Treated obs =	2,340	16,871.3
Control obs =	31,394	16,862.7

Piperacillin/tazobactam:

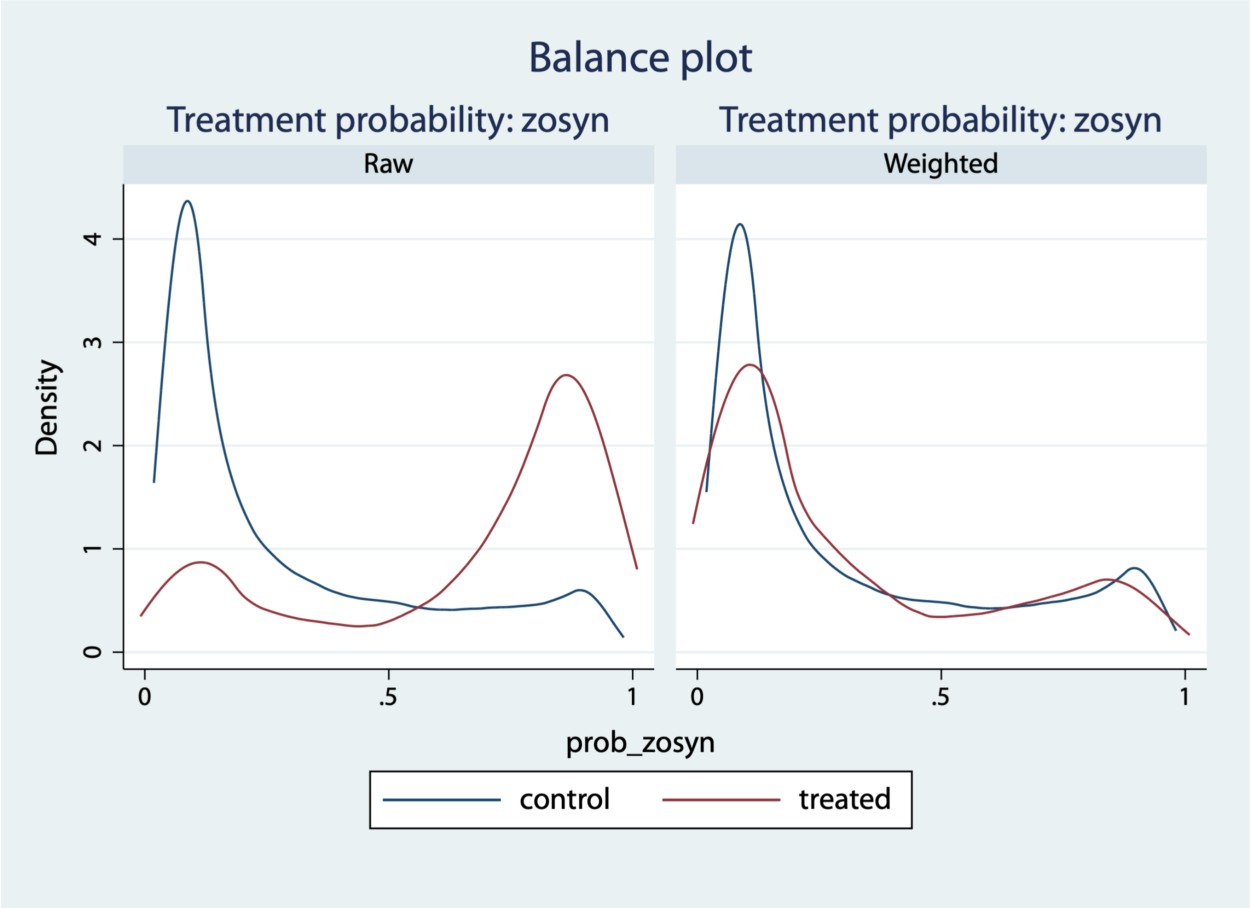

Covariate balance summary:

		Raw	Weighted
Number of obs	=	26,960	26,960.0
Treated obs	=	1,937	13,489.3
Control obs	=	25,023	13,470.7

Vancomycin:

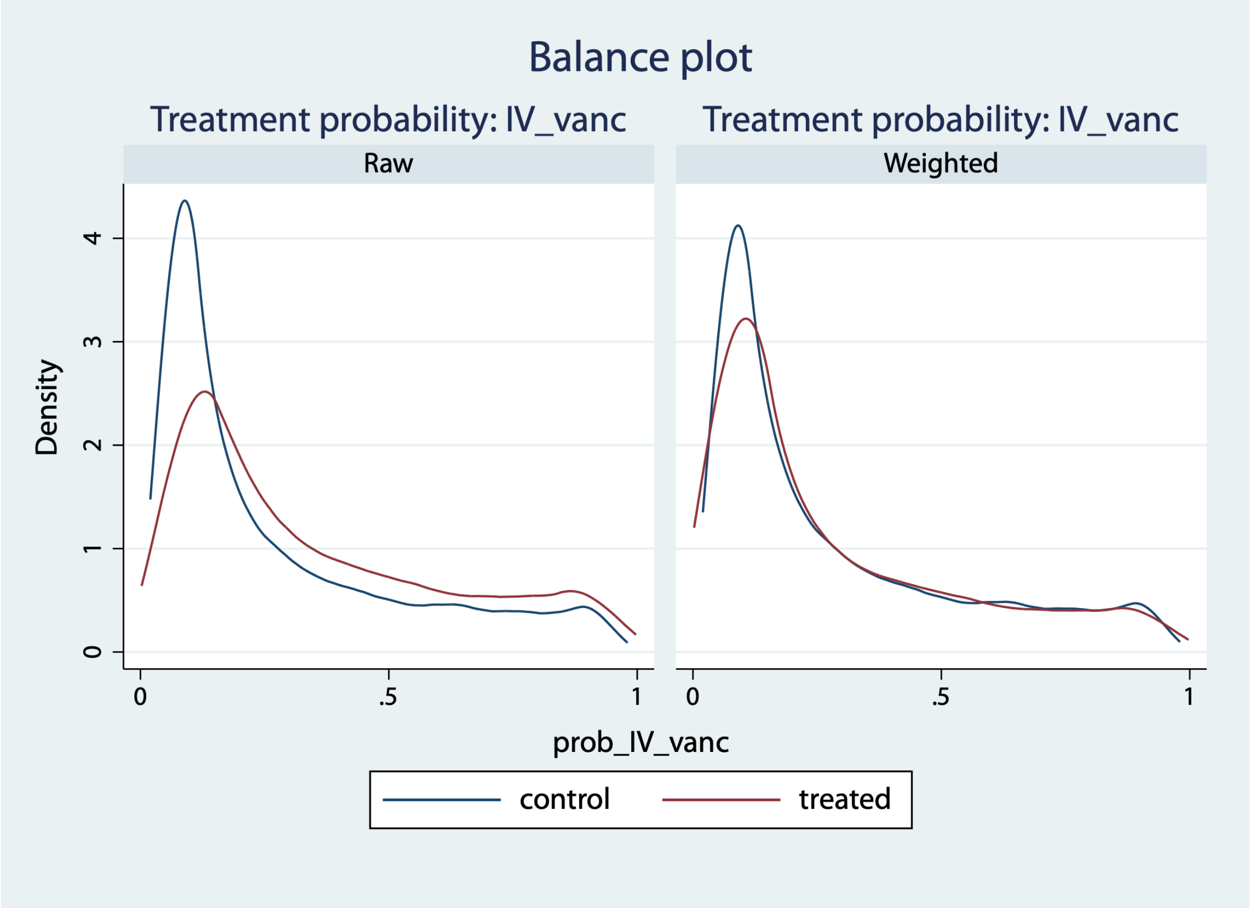

Covariate balance summary:

	Raw	Weighted
Number of obs =	19,926	19,926.0
Treated obs =	3,050	9,964.2
Control obs =	16,876	9,961.8

7. Post-hoc analysis comparing azithromycin against no antibiotics

The results of our comparison between receipt of azithromycin and no azithromycin—which suggested that azithromycin *decreased* the rate of CDI—were discordant with theory and previous analyses. To better understand why our analysis produced this result, we then limited our dataset to patients who did not receive doses of any other antimicrobials (ceftriaxone, cefepime, piperacillin/tazobactam, meropenem, levofloxacin, or parenteral vancomycin).

In that more limited dataset, we again fit a three-level logistic regression model predicting receipt of azithromycin, compared to no antibiotics of interest, using the same approach in our primary analyses. To achieve convergence, this model required the simplification of reducing the knots in two of our continuous variables: hospital_day and WBC_count. In the model form that converged, each of those two variables had three knots rather than the default used in our primary analysis (4 knots each).

We again limited the dataset to patients at intermediate probability of receipt (between 5% and 95%), and again fit an AIPW model. However, after these additional exclusions, there were too few outcome events (6) to include any predictors in an outcome model. This therefore simplifies from an AIPW to an IPW model.

In this model, the average treatment effect of one daily dose-equivalent of azithromycin was -2.6e-06 ($p>0.2$, 95% CI: -0.0001 to 0.0001). Our covariate balance plot is shown below:

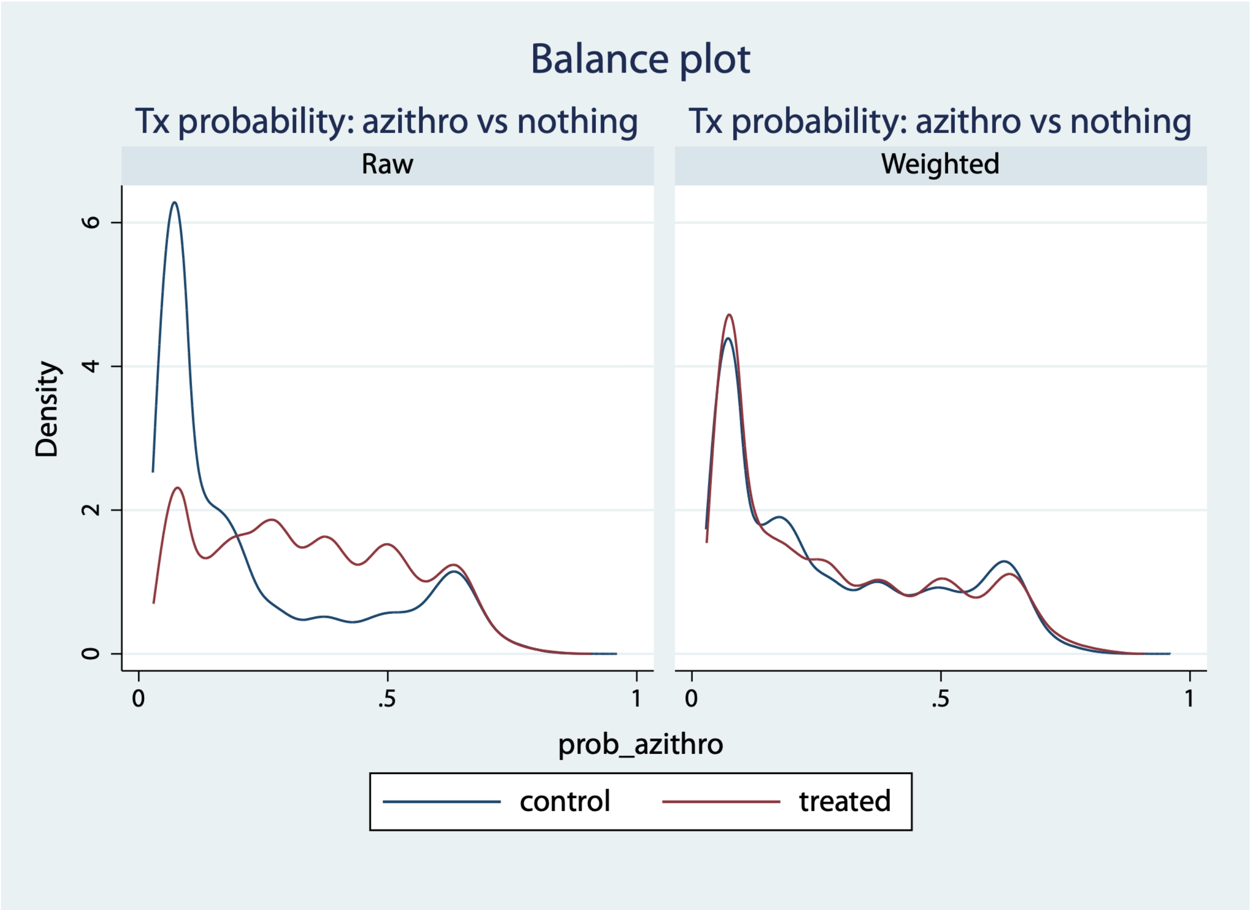

Because azithromycin appeared to reduce the risk of CDI compared to not receiving azithromycin, but did not appear to have an effect when compared to no antibiotics, we conclude that in this dataset azithromycin was likely given (in part) as a less-risky alternative to other antimicrobials.

Note that we designed this analysis based on our earlier results; it should therefore be considered somewhat exploratory.

8. Results of sensitivity analyses with inclusion/exclusion at 24 or 72 hours after admission, rather than 48

24 hours:

	Absolute risk increase	95% CI		p
**Azithromycin**	−0.0025	−0.0033	−0.0018	<0.001
**Ceftriaxone**	−0.0009	−0.0017	−0.0002	0.016
**Cefepime**	0.0030	0.0000	0.0060	0.046
**Piperacillin/tazobactam**	0.0029	−0.0023	0.0081	>0.2
**Vancomycin**	0.0090	0.0048	0.0133	<0.001

72 hours:

	Absolute risk increase	95% CI		p
**Azithromycin**	−0.0031	−0.0041	−0.0020	<0.001
**Ceftriaxone**	−0.0011	−0.0023	0.0000	0.052
**Cefepime**	0.0060	0.0002	0.0117	0.042
**Piperacillin/tazobactam**	0.0031	−0.0022	0.0085	>0.2
**Vancomycin**	0.0082	0.0045	0.0118	<0.001
